# Capture of organic iodides from nuclear waste by metal-organic framework-based molecular traps

**DOI:** 10.1038/s41467-017-00526-3

**Published:** 2017-09-07

**Authors:** Baiyan Li, Xinglong Dong, Hao Wang, Dingxuan Ma, Kui Tan, Stephanie Jensen, Benjamin J. Deibert, Joseph Butler, Jeremy Cure, Zhan Shi, Timo Thonhauser, Yves J. Chabal, Yu Han, Jing Li

**Affiliations:** 1 Department of Chemistry and Chemical Biology, Rutgers University, Piscataway, NJ 08854 USA; 2 Advanced Membranes and Porous Materials Center, Physical Sciences and Engineering Division, King Abdullah University of Science and Technology, Thuwal, 23955-6900 Saudi Arabia; 3 State Key Laboratory of Inorganic Synthesis and Preparative Chemistry, College of Chemistry, Jilin University, Changchun, 130012 People’s Republic of China; 4 Department of Materials Science and Engineering, University of Texas at Dallas, 800W Campbell Rd., Richardson, TX 75080 USA; 5 Department of Physics, Wake Forest University, Winston-Salem, NC 27109 USA; 6 Department of Chemistry, Massachusetts Institute of Technology, Cambridge, MA 02139 USA

## Abstract

Effective capture of radioactive organic iodides from nuclear waste remains a significant challenge due to the drawbacks of current adsorbents such as low uptake capacity, high cost, and non-recyclability. We report here a general approach to overcome this challenge by creating radioactive organic iodide molecular traps through functionalization of metal-organic framework materials with tertiary amine-binding sites. The molecular trap exhibits a high CH_3_I saturation uptake capacity of 71 wt% at 150 °C, which is more than 340% higher than the industrial adsorbent Ag^0^@MOR under identical conditions. These functionalized metal-organic frameworks also serve as good adsorbents at low temperatures. Furthermore, the resulting adsorbent can be recycled multiple times without loss of capacity, making recyclability a reality. In combination with its chemical and thermal stability, high capture efficiency and low cost, the adsorbent demonstrates promise for industrial radioactive organic iodides capture from nuclear waste. The capture mechanism was investigated by experimental and theoretical methods.

## Abstract (web summary)

Capturing radioactive organic iodides from nuclear waste is important for safe nuclear energy usage, but remains a significant challenge. Here, Li and co-workers fabricate a stable metal–organic framework functionalized with tertiary amine groups that exhibits high capacities for radioactive organic iodides uptake.

## Introduction

Currently, nuclear power provides ~11% of the world’s electricity offering a cost-effective option compared to other energy sources^1^. Rapidly increasing global energy needs will likely increase the demand for nuclear energy in the future. Under normal operating conditions, fuel rods used in nuclear power plants need to be reprocessed. This procedure involves the production of complex off-gas mixtures consisting of HNO_3_, NO_2_, and N_2_O_5_ along with radioactive molecular iodine (I_2_) and organic iodides (ROIs, e.g., methyl iodide and ethyl iodide) at elevated temperatures (e.g., 150 °C)^2–4^. Radioactive I_2_ and ROI species must be selectively captured and sequestered to ensure safe nuclear energy usage. ROI species are known to be particularly difficult to capture, and a recent study has shown that the CH_3_I adsorption rate is only half of that for I_2_ in the case of Ag@MOR^5^. It thus tends to leak into the environment more easily^6^. Among various current capture technologies, solid sorbent-based fixed-bed methods have proven superior due to their simplicity and relatively low cost^7^. Examples of solid adsorbents for capturing ROIs from off-gas mixtures include triethylenediamine (TED) impregnated activated carbon (AC)^8, 9^, and silver impregnated/exchanged solid supports such as silica, alumina, and zeolites^10–14^. Typically the temperature of the off-gas is performed at high temperature (such as ~150 °C) in order to accelerate the chemical reactions and to remove adsorbed water from the narrow pores of the supports^15^. AC-based adsorbents, however, can only be used under 120 °C and are limited to specific applications absent of NO_x_^16^ because of low ignition temperatures and the risk of formation of explosive compounds. Silver functionalized porous materials are capable of performing at higher temperatures, but the high costs associated with the noble metal limits their widespread application. Additionally, much to their detriment, chemical adsorption of CH_3_I—necessary for its high uptake at high temperature in such systems—makes the silver-based adsorbents poorly recyclable. Based on these considerations, and the fact that the uptake capacity of all existing adsorbents remains insufficient, new types of adsorbent materials that are noble metal free, highly efficient, cost effective, recyclable, and safe to use, are much needed for ROIs capture.

To tackle the aforementioned challenges, a desired ROI adsorbent must possess the following features: extraordinarily high adsorption capacity at the reprocessing temperature; high tolerance toward nitrogen-based oxides, acidity, and humidity; high thermal stability (≥150 °C) that meets the required reprocessing conditions; high efficiency well above reprocessing facility regulatory requirements and low cost and excellent recyclability. To fulfill this goal, we investigate a different type of crystalline porous materials, metal-organic frameworks (MOFs)^17–27^, as tunable and recyclable solid adsorbents for ROIs capture. We reason that such materials can potentially offer the following advantages: large and adjustable surface area and pore size enable accommodation of a large amount of ROI molecules and thus result in high ROI capacity^28–38^; modular nature allows for rational design and tailoring of structural topology and functional sites^39–42^; multivariate syntheses offer possibilities for obtaining topologically identical yet functionally diverse crystalline frameworks^43, 44^; modifiable open metal sites (OMSs) that form reversible coordination bonds with tertiary amines provide an effective means for recyclability^45–47^. While numerous previous investigations have illustrated that MOFs serve as an excellent platform for capture of radioactive molecular iodine^32–35, 48^, their use as adsorbent materials to effectively trap ROI species remains unexploited to this date.

Here we demonstrate that highly efficient ROI molecular traps can be obtained via tertiary amine grafting to binding sites within a MOF framework. One such designed molecular trap, MIL-101-Cr-TED, exhibits an exceptionally high uptake capacity of 71 wt% for methyl iodide at 150 °C. Under identical capture conditions, this performance is more than 340% higher than that of the industrial adsorbent Ag^0^@MOR, a leading material in the United States for ROIs capture^10, 49^. Furthermore, the pristine MOF sample can be regenerated and reused multiple times without decrease in uptake capacity. This is a notable advancement as high-temperature recyclable adsorbent materials have been pursued since the 1980s but with very little success to this date. Coupled with its high chemical and thermal stability, relatively low cost, and high capture efficiency at both room and high temperatures, amine functionalized MIL-101-Cr-TED demonstrates a considerable potential for use as MOF-based adsorbent for the ROIs capture technology. Employing both experimental and theoretical methods, we further carried out an in-depth study to investigate and understand the mechanism of CH_3_I capture in the MIL-101-Cr-TED system. Our findings show that the construction and optimization of such molecular traps can be expanded to other MOFs by a suitable combination of robust frameworks with strong binding sites and tertiary amine molecules, which may lead to a large number of ROI capture materials. This strategy thus paves the way for further research and advancement on MOF-based molecular traps for their ultimate utility in ROIs capture from nuclear waste.

## Results

### Synthesis and characterization

**Fig. 1:**
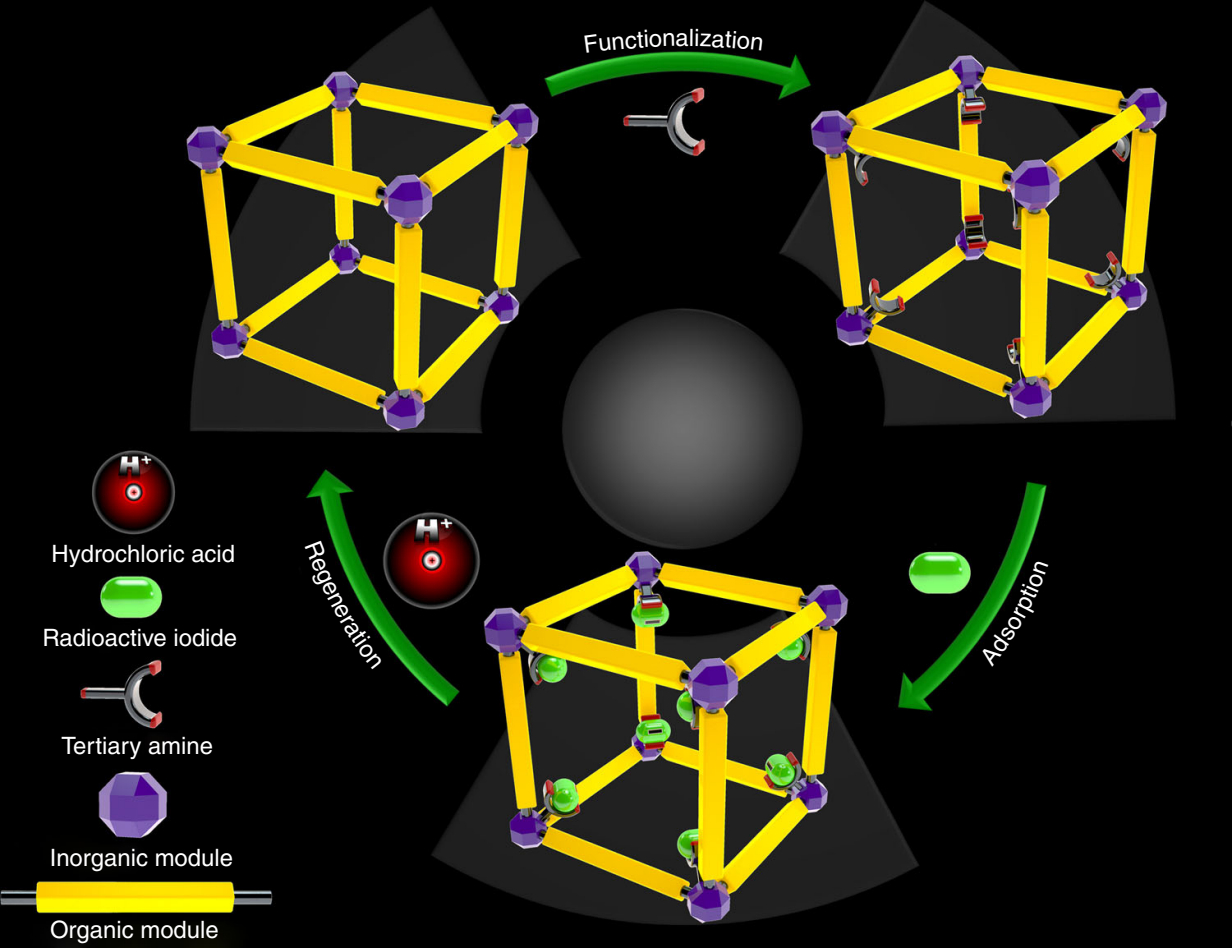
The design strategy. A schematic illustrating the design of a recyclable MOF molecular trap for effective capture of radioactive organic iodides from nuclear waste

To construct high performance molecular traps (Fig. 1) that are highly stable, efficient, and recyclable for capturing organic iodides, we chose MIL-101-Cr as a model support material because of its large surface area (>3300 m^2^ g^−1^), high acid and moisture stability, thermal stability (~ 300 °C), and relatively low cost^44^. Two tertiary amines, triethylenediamine (TED) and hexamethylenetetramine (HMTA), were selected as functional molecules for post-synthetic modification of the MOF framework. Both species can use a single nitrogen to bind to the OMSs on the Cr trinuclear secondary building unit of MIL-101-Cr^45, 47, 50, 51^, with the remaining nitrogen atoms available as binding sites for organic iodides (Supplementary Fig. 1). MIL-101-Cr-TED and MIL-101-Cr-HMTA were obtained by stirring MIL-101-Cr with TED or HMTA in benzene or chloroform at 110 °C for 24 h in a resealed flask under nitrogen atmosphere. Transmission electron microscopy (TEM) images (Supplementary Fig. 2) of both functionalized MOFs show similar crystal morphology when compared to as-made MIL-101-Cr, suggesting retention of crystallinity and morphology after amine functionalization.

**Fig. 2:**
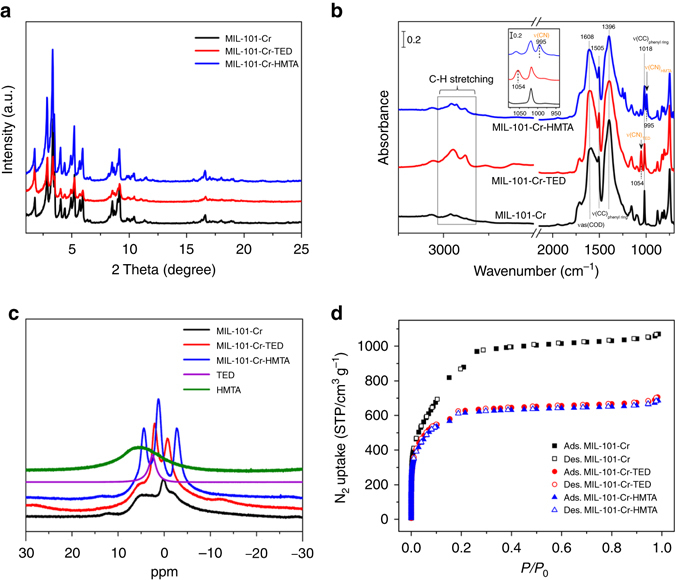
Characterization of MIL-101-Cr and tertiary amine functionalized MIL-101-Cr. **a** PXRD patterns of MIL-101-Cr, MIL-101-Cr-TED, and MIL-101-Cr-HMTA. **b** FT-IR spectra of MIL-101-Cr, MIL-101-Cr-TED, and MIL-101-Cr-HMTA collected on samples after dehydration at 150 °C, the *inset* shows the mode associated with the C-N stretching vibration, at 1054 cm^−1^ in MIL-101-Cr-TED and 995 cm^−1^ in MIL-101-Cr-HMTA, based on refs ^51, 52^. Note that the weak band at 1059 cm^−1^ in MIL-101-Cr-HMTA spectrum is due to the CNC deformation vibration. The assignment of MOF phonon modes are based on ref. ^53^. **c** Solid ^1^H NMR spectra of MIL-101-Cr, TED, HMTA, MIL-101-Cr-TED, and MIL-101-Cr-HMTA. **d** N_2_ sorption isotherms of MIL-101-Cr, MIL-101-Cr-TED, and MIL-101-Cr-HMTA collected at 77 K

The successful grafting of tertiary amine groups onto MIL-101-Cr was confirmed by powder X-ray diffraction (PXRD), Fourier transform infrared (FT-IR) spectroscopy, X-ray photoelectron spectroscopy (XPS), solid-state ^1^H NMR, and elemental analysis. PXRD analysis shows that the diffraction profiles are unchanged after amine functionalization (Fig. 2a), indicating that the crystal structure of the functionalized material remains intact. The IR absorption spectra in Fig. 2b shows two additional peaks (at 1054 and 995 cm^−1^) associated with TED and HMTA that are not present in pristine MIL-101-Cr. This amine-associated mode (see *inset* of Fig. 2b) is attributed to the skeletal C-N stretching mode^52, 53^, confirming successful grafting. In addition, new features between 3000 and 2800 cm^−1^ are assigned to the aliphatic C-H stretching modes of TED and HMTA molecules (Fig. 2b)^52, 53^. XPS spectra (Supplementary Fig. 3) confirm that the N(1s) core level in MIL-101-Cr-TED and MIL-101-Cr-HMTA is at a binding energy (~400.0 eV) consistent with the nitrogen within tertiary amine groups^54^. Solid-state ^1^H NMR studies identify chemical shifts at 2.1 and 1.3 ppm for the hydrogen of –CH_2_– groups in TED and HMTA (Fig. 2c)^55^. These observations establish that TED and HMTA are chemically incorporated onto the OMSs. Elemental analysis reveals a nitrogen content of 6.38 and 12.15 wt% for MIL-101-Cr-TED and MIL-101-Cr-HMTA, respectively. This is equivalent to ~2/3 TED or HMTA molecules grafted to each Cr, which is consistent with the previous report of two available open metal sites on each Cr_3_O cluster (2:3)^45^.

Nitrogen gas adsorption-desorption isotherms collected at 77 K indicate that the modification of TED and HMTA molecules onto MIL-101-Cr leads to a decrease in the Brunauer–Emmett–Teller surface area from 3342 to 2282 m^2 ^g^−1^ and 2272 m^2^ g^−1^ for MIL-101-Cr-TED and MIL-101-Cr-HMTA, respectively (Fig. 2d). Despite such decreases, the surface areas of the two amine-functionalized samples are significantly higher than any other benchmark porous materials, which usually exhibit moderate surface areas of ~300–1000 m^2 ^g^−1^ ^10–14^. Based on the pore size distribution (Supplementary Fig. 4), both MIL-101-Cr-TED and MIL-101-Cr-HMTA have pore diameters of about 16 and 21 Å, which are large enough for effective mass transfer during nuclear waste reprocessing. Thermogravimetric analysis (TGA) shows that MIL-101-Cr-TED and MIL-101-Cr-HMTA are stable up to 260 and 220 °C, respectively (Supplementary Note 1 and Supplementary Fig. 5). Isothermal TG analysis of amine grafted MIL-101-Cr samples clearly show that both TED and HMTA remain attached to the framework without losing mass upon prolonged heating at 150 °C for 12 h (Supplementary Fig. 6). The high thermal stability of the two compounds enables their use at the elevated working temperature (such as 150 °C) required for nuclear waste treatment. This result is consistent with the high binding energies of TED and HMTA to the OMSs (Supplementary Note 2, Supplementary Fig. 7, and Supplementary Table 1) obtained from ab initio theoretical calculations. The calculations also show that the TED and HMTA bind significantly more strongly to the OMSs compared to H_2_O molecules, further suggesting the feasibility of their application under humid conditions. In addition, two materials also show excellent stability in both CH_3_I gas stream and simulated off-gas mixture, as evident from the PXRD patterns collected after the experiments (Supplementary Fig. 8). The large surface area coupled with the high thermal and chemical stability of both MIL-101-Cr-TED and MIL-101-Cr-HMTA prompted us to evaluate their performance as molecular traps for the removal of ROIs from off-gas mixtures.

### Radioactive organic iodides sorption studies

To evaluate the performance of MIL-101-Cr-TED and MIL-101-Cr-HMTA for ROIs capture, as-made samples (~20 mg of each) were placed in a thermogravimetric analyzer and a CH_3_I steam with partial pressure of 0.2 atm was passed through the sample cell using N_2_ as a carrier gas. The adsorption amount was monitored by recording sample mass as a function of time. As shown in Supplementary Fig. 9, MIL-101-Cr-TED and MIL-101-Cr-HMTA rapidly absorb 120 and 136 wt% CH_3_I within 10 min at 30 °C and reach their maximum uptake amount of 160 and 174 wt% by 120 min, respectively. The absorption amounts are significantly higher than all benchmark materials used for CH_3_I adsorption under the same conditions, such as TED- and HMTA-impregnated activated carbon (TED@AC and HMTA@AC) and silver functionalized zeolites (including ZSM-5, 13X, and mordenite (Ag^+^@ZSM-5, Ag^+^@13X, Ag^+^@MOR, and Ag^0^@MOR) with CH_3_I uptake amounts of 52, 54, 28, 45, 29, and 25 wt%, respectively (Supplementary Fig. 10)).

**Fig. 3:**
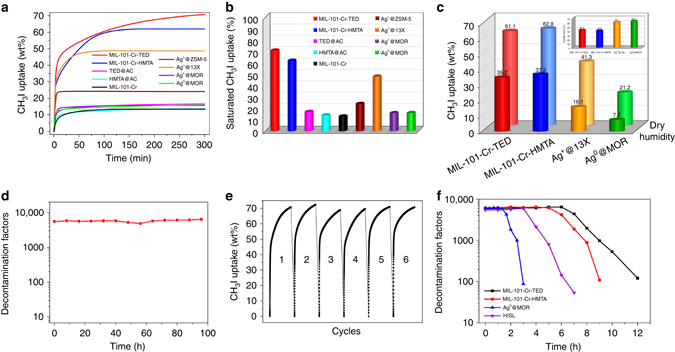
The CH_3_I capture performance. **a** Sorption isotherms of CH_3_I in MIL-101-Cr-TED, MIL-101-Cr-HMTA, and selected benchmark sorbent materials at 150 °C with partial pressure of 0.2 atm for CH_3_I. **b** Comparing the saturated CH_3_I uptake in MIL-101-Cr-TED, MIL-101-Cr-HMTA, and selected benchmark sorbent materials at 150 °C with partial pressure of 0.2 atm for CH_3_I. **c** The CH_3_I uptake at 150 °C under dry and humidity (RH = 81%) conditions by breakthrough experiment (*back row*: dry conditions; *front row*: humid conditions), (*insert*) the uptake drop ratio by comparing the CH_3_I uptake of dry and humid conditions. **d** Decontamination factors of CH_3_I by MIL-101-Cr-TED under simulated conditions representing gas mixtures produced during CH_3_I reprocessing, which include CH_3_I (50 ppm), H_2_O, HNO_3_, NO_2_, and NO at 150 °C. **e** The recyclability of MIL-101-Cr-TED for CH_3_I capture. **f** Decontamination factors of total iodine (CH_3_I and I_2_) by MIL-101-Cr-TED, MIL-101-Cr-HTMA, and comparable samples Ag^0^@MOR and HISL under the simulated conditions of an off-gas mixture: I_2_ (150 ppm), CH_3_I (50 ppm), H_2_O, HNO_3_ and NO_x_ at 150 °C

Since the capture of organic iodides from off-gas mixtures is usually performed at elevated temperatures (e.g., ~150 °C)^15^, we tested the CH_3_I uptake capacity at 150 °C for both samples and several benchmark materials. MIL-101-Cr-TED and MIL-101-Cr-HMTA can fast adsorb 48 and 39 wt% CH_3_I within 20 min (Fig. 3a). For the same time period, Ag^0^@MOR (the industrial zeolite material) only shows a CH_3_I uptake of 13 wt% under the same conditions. The fast adsorption rate of MIL-101-Cr-TED and MIL-101-Cr-HMTA is an important feature for practical applications of radioactive CH_3_I capture. The kinetic constants of MIL-101-Cr-TED and MIL-101-Cr-HMTA were calculated to be 0.30 and 0.36, respectively (Supplementary Note 3 and Supplementary Table 2), based on Lagergren’s pseudo first-order kinetic model. These values are similar as of other comparable materials, which have kinetic constant values between 0.19 and 0.62 (Supplementary Table 2 and Supplementary Fig. 11). At this temperature, the maximum uptake amounts at 0.2 atm are 71and 62 wt% for MIL-101-Cr-TED and MIL-101-Cr-HMTA, respectively (Fig. 3a). These values are 4.4 and 3.9 times that of Ag^0^@MOR (16 wt%), a leading adsorbent material for capturing ROIs in the US nuclear fuel reprocessing industry^48^. We also compared the performance of MIL-101-Cr-TED with Ag^+^@13X, a zeolite with the highest silver content. Ag^+^@13X has a higher CH_3_I uptake amount (max. 48 wt%) compared to Ag^0^@MOR, but its low-acid resistance presents a serious drawback. MIL-101-Cr-TED adsorbs 1.5 times more CH_3_I than Ag^+^@13X under identical conditions. The capture capacity of MIL-101-Cr-TED is also much higher than the other benchmark materials, such as TED@AC, HMTA@AC, Ag^+^@ZSM-5, and Ag^+^@MOR, with uptake amounts of 17, 14, 24, and 16 wt%, respectively (Fig. 3b). Compared to the performance of pristine MIL-101-Cr (CH_3_I uptake: 13 wt%), TED functionalization leads to a remarkable increase of ~5.5 times. Based on these comparisons, MIL-101-Cr-TED clearly ranks as the top candidate for adsorbent-based capture and removal of ROIs during nuclear fuel reprocessing. The exceptionally high uptake capacity of tertiary amine functionalized MIL-101-Cr can be attributed to two main factors: (a) relatively high surface area of the adsorbent after functionalization, and more importantly, (b) effective grafting of TED and HMTA onto the MOF pore surface (via OMSs) creating molecular traps that offer greatly enhanced bonding interactions toward organic iodides. In addition, we performed adsorption experiments on two other organic iodides, CH_3_CH_3_I and CH_3_CH_2_CH_2_I. High uptake amounts were achieved for both species: 75 and 51 wt% of CH_3_CH_3_I and 74 and 54 wt% of CH_3_CH_2_CH_3_I in MIL-101-Cr-TED and MIL-101-Cr-HMTA, respectively (Supplementary Fig. 12).

To assess the influence of humidity on the organic iodide uptake capacity, a crucial aspect for a sorbent’s performance metrics in real-world applications, we performed column breakthrough tests at 150 °C under both dry and humid conditions (RH = 81% at 23 °C) (Supplementary Figs. 13–16 and Supplementary Table 3). For Ag^+^@13X, the zeolite with the best performance based on our parallel experimental results, the uptake drops from 41.3 to 16.1 wt%, a significant decrease of 61% (Fig. 3c, *insert*). We also observed a dramatic decrease of 63.7% for Ag^0^@MOR, with an uptake of 21.2 wt% under dry conditions and 7.7 wt% under humid conditions. For MIL-101-Cr-TED and MIL-101-Cr-HMTA, however, the extent of decrease is much smaller (42.4 and 40.8%, respectively). Based on the breakthrough data, the uptake capacities of MIL-101-Cr-TED and MIL-101-Cr-HMTA are 2.2 and 2.3 times higher than that of Ag^+^@13X, and 4.6 and 4.8 times greater than Ag^0^@MOR at 150 °C under humid conditions, underscoring the robustness of MIL-101-Cr-TED and MIL-101-Cr-HMTA adsorbents in humid environments. The higher hydrophilicity of zeolites compared to MOF materials may account for this difference, which makes MOFs are an advantageous platform for CH_3_I capture over conventional silver functionalized zeolites. As in the cases of previous studies^8^, moisture will affect the performance of capture materials. The decrease in the CH_3_I uptake capacity under humidity is likely due to the fact that a large amount of water molecules will take up the chemisorption sites and porous space, thereby reducing the overall loading amount.

Nuclear processing facilities’ regulatory standards require a “decontamination factor” (DF) of 3000 (99.967% of active species removed) for CH_3_I reprocessing, which is defined as the ratio of radioactivity before and after decontamination procedures^56^. To evaluate the efficiency of MIL-101-Cr-TED as a molecular trap for capturing CH_3_I from nuclear waste, we performed breakthrough experiments under conditions simulating the gas mixtures produced during CH_3_I reprocessing, which include H_2_O, HNO_3_, NO_2_, and NO at 150 °C^56^. As shown in Fig. 3d, for a very low CH_3_I concentration of 50 ppm that mimics real-world off-gas reprocessing conditions, very high DFs for MIL-101-Cr-TED are achieved, with the values range between 4800 and 6300, which are substantially higher than the reprocessing facility regulatory requirements^57^. This means that about 99.979–99.984% CH_3_I can be removed by MIL-101-Cr-TED under such conditions. The result further illustrates that the MOF-based molecular traps are highly suitable for ROIs capture from nuclear waste off-gas mixtures.

After CH_3_I adsorption, pristine MIL-101-Cr can be regenerated by washing with 3 M HCl followed by ethanol solution, resulting in complete recovery of the pristine MOF sample (Supplementary Figs. 17 and 18). The framework can then be re-functionalized to MIL-101-Cr-TED or MIL-101-Cr-HMTA following the initial procedure. The regenerated MIL-101-Cr-TED retains ~97–100% of the original loading capacity during the five full cycles (Fig. 3e). As silver-based adsorbents often suffer from a dramatic loss of adsorption capacity after only a few cycles (e.g., 50% loss for Ag^+^@13X after five cycles), our investigation thus established a recyclable system for ROIs capture from nuclear waste that is unattainable by any other known high-temperature adsorbents. We also estimated the CH_3_I capture cost for MIL-101-Cr-TED and Ag^0^@MOR. As shown in Supplementary Table 4, for each cycle the cost for the latter is 35 times of the former, a significant saving for MIL-101-Cr-TED.

To evaluate the material capability of capturing ROIs under the real-world conditions, we also conducted breakthrough experiments (Fig. 3f and Supplementary Fig. 19) at the conditions that mimic an off-gas mixture with high humidity (RH = 95%) and in the presence of HNO_3_ and NO_x_. The mixture also contains both radioactive species I_2_ (150 ppm) and CH_3_I (50 ppm). At 150 °C, the total iodine uptake amounts calculated based on unit weight and unit volume, as well as packing density of the sorbents (Supplementary Fig. 20) are summarized in Supplementary Table 5. The uptake are 38 and 33 wt% for MIL-101-Cr-TED and MIL-101-Cr-HMTA, respectively, and high DF values (>5000) are obtained (Fig. 3f and Supplementary Table 5). These values are significant higher than those of Ag^0^@MOR (5 wt%)^12^ and pure silica zeolite HISL (16 wt%)^15^ under the same conditions (Fig. 3f and Supplementary Table 5). The results suggest that amine grafted MIL-101-Cr materials are capable of effectively capturing CH_3_I in off-gas mixtures containing I_2_. In addition, I_2_ adsorption isotherms were collected under similar experimental conditions (150 °C and 150 ppm) and its interaction with amine functionalized MIL-101-Cr were evaluated (Supplementary Note 4 and Supplementary Figs. 21–23). The possible adsorption mechanisms in simulated gas mixture were also investigated and binding energies were estimated by DFT calculations (Supplementary Note 2, Supplementary Fig. 24, and Supplementary Table 6). Similar experiments were also performed at room temperature, which gives the same trend (Supplementary Table 5). Furthermore, recyclability tests of MIL-101-Cr-TED under simulated off-gas conditions verified that the total iodine uptake remains the same after three cycles (Supplementary Fig. 25).

### Investigation of CH_3_I-binding interactions mechanisms

The outstanding performance of MIL-101-Cr-TED and MIL-101-Cr-HMTA encouraged us to investigate the possible interaction mechanisms between CH_3_I molecules and the MOF host. Gas adsorption experiments carried out at 150 °C show evidence of both physisorption and chemisorption for CH_3_I (Supplementary Fig. 26), but chemisorption is the dominant mode of interaction (76 and 68% for MIL-101-Cr-TED and MIL-101-Cr-HMTA, respectively) at that temperature, with most CH_3_I molecules being chemically trapped within the framework. The chemisorbed amounts are 54 and 43 wt%, respectively, for MIL-101-Cr-TED and MIL-101-Cr-HMTA, and physisorbed amounts, 17 and 19 wt%, respectively. The partial pressure of CH_3_I shows very little influence on the chemisorption capacity of the sorbents (Supplementary Fig. 27).

**Fig. 4:**
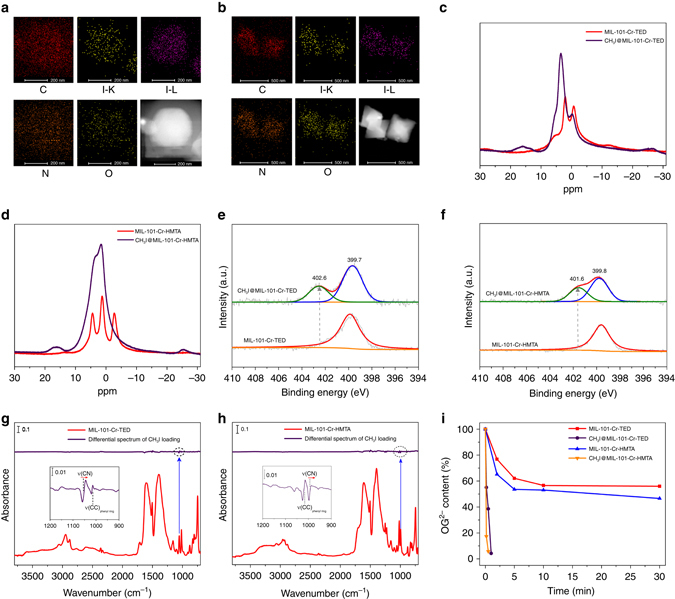
The mechanism of CH_3_I capture by MIL-101-Cr-TED and MIL-101-Cr-HMTA. **a** The elemental mapping of CH_3_I loaded MIL-101-Cr-TED. **b** The elemental mapping of CH_3_I loaded MIL-101-Cr-HMTA. **c** Solid ^1^H NMR spectra of MIL-101-Cr-TED and CH_3_I@MIL-101-Cr-TED. **d** Solid ^1^H NMR spectra of MIL-101-Cr-HMTA and CH_3_I@MIL-101-Cr-HMTA. **e** XPS spectra of N(1s) for MIL-101-Cr-TED and CH_3_I@MIL-101-Cr-TED (*gray*, experiment curves; *red*, *blue*, and *green*: fitted curves; *orange*: baselines). **f** XPS spectra of N(1s) for MIL-101-Cr-HMTA and CH_3_I@MIL-101-Cr-HMTA (*gray*, experiment curves; *red*, *blue*, and *green*: fitted curves; *orange*: baselines). **g** In situ IR spectra (*green*) of ~150 Torr CH_3_I exposed MIL-101-Cr-TED referenced to the activated MIL-101-Cr-TED; and IR absorption spectra (*red*) of activated MIL-101-Cr-TED referenced to KBr pellet in vacuum (<20 mtorr). **h** In situ IR spectra (*green*) of ~150 Torr CH_3_I exposed MIL-101-Cr-HMTA referenced to the activated MIL-101-Cr-HMTA; and IR absorption spectra (*red*) of activated MIL-101-Cr-HMTA referenced to KBr pellet (<20 mtorr). **i** Ion exchange efficiencies of an anionic dye (Orange G or OG) by pristine MIL-101-Cr-TED and MIL-101-Cr-HMTA and functionalized CH_3_I@MIL-101-Cr-TED and CH_3_I@MIL-101-Cr-HMTA as a function of time

The strong interaction between CH_3_I molecules and MIL-101-Cr-TED and MIL-101-Cr-HMTA was verified by HRTEM-EDS, solid-state ^1^H NMR, XPS, and in situ FT-IR studies. Elemental mapping using HRTEM-EDS confirms uniformly dispersed iodine (CH_3_I) within the crystal samples of CH_3_I-loaded MIL-101-Cr-TED and MIL-101-Cr-HMTA (Fig. 4a, b). Solid-state ^1^H NMR spectra of CH_3_I-loaded MIL-101-Cr-TED and MIL-101-Cr-HMTA show a new peak at ~16 ppm (Fig. 4c, d), which can be assigned to the H atoms of CH_3_I^58^. The XPS data are consistent with this interpretation, as a good fraction of the N(1s) core level is shifted to higher binding energies upon CH_3_I loading (402.6 and 401.6 eV for MIL-101-Cr-TED and MIL-101-Cr-HMTA, respectively) as compared with the as-synthesized analogs. This shift indicates that the valence of N is increased by interacting with guest CH_3_I molecules (Fig. 4e, f). The N(1s) core level shifts more in CH_3_I@MIL-101-Cr-TED, 2.9 eV, compared to 1.8 eV in CH_3_I@MIL-101-Cr-HMTA, indicating less charge transfer in CH_3_I@MIL-101-Cr-HMTA. Consistent with this interaction, the I 3d_5/2_ peak of CH_3_I, positioned at 620.2 eV for the molecular adsorbed state, was red shifted to 618.7 ± 0.1 eV in MIL-101-Cr-TED and to 618.6 ± 0.1 eV in MIL-101-Cr-HMTA (Supplementary Fig. 28). This value is typical of CH_3_I dissociatively adsorbed on metal or metal oxide surfaces as previously observed (e.g., Ni(100), TiO_2_)^59, 60^. Similarly, a characteristic shift (~1 cm^−1^) of the signature ν(C-N) vibrational mode is observed with FT-IR upon CH_3_I loading of MIL-101-Cr-TED and MIL-101-Cr-HMTA (Fig. 4g, h). These results suggest the formation of strong chemical bonds between tertiary amines and CH_3_I, yielding ionic species (R_3_N-CH_3_)^+^ I^−^ at high temperatures (Supplementary Fig. 29).

To test this hypothesis, we performed an ion-exchange experiment by using an anionic organic dye as a molecular probe. UV-vis measurements were used to monitor the adsorption efficiency of Orange G (OG) dye by the pristine MIL-101-Cr-TED and MIL-101-Cr-HMTA, as well as CH_3_I-loaded MIL-101-Cr-TED and MIL-101-Cr-HMTA (Fig. 4i and Supplementary Fig. 30). Fast anion-exchange kinetics were observed when using CH_3_I-loaded MIL-101-Cr-TED and MIL-101-Cr-HMTA as adsorbents, which adsorb over 95% of the anionic organic dye within 1 min and 30 s, respectively. In contrast, pristine MIL-101-Cr-TED and MIL-101-Cr-HMTA samples adsorb only 44 and 54% of the OG and over a much longer time period of 30 min. The fast kinetics of CH_3_I-loaded samples confirms the formation of (R_3_N-CH_3_)^+^ I^−^ in high concentration when reacting CH_3_I with the free nitrogen atom of the amine molecules grafted on MIL-101-Cr-TED and MIL-101-Cr-HMTA at high temperature. In addition, the formation of I^−^ ions after CH_3_I sorption was verified by AgNO_3_ titration of dye exchanged filtrates, which yielded AgI precipitate (Supplementary Fig. 31).

To better understand the relationship between the chemisorption and physisorption binding regime at different temperatures, we conducted simulations of the corresponding binding mechanisms (Supplementary Table 7). In particular, we performed an ab initio transition-state search to find the pathway that connects the two regimes and the energy barrier that separates them (see Supplementary Fig. 32). We find that an energy barrier of 459 meV separates the physisorption and chemisorption case while the energy difference for the products is 200 meV higher than that of the reactants (Supplementary Fig. 32). This energy difference is small enough at room temperature to allow some ionic I^−^ to be present in the system, but large enough to prevent most CH_3_ from having covalently bonding to the nitrogen in TED. This finding is also consistent with our experimental results (Supplementary Figs. 33 and 34). Once the temperature increases to 150 °C, enough energy enters the system to overcome the energy barrier, resulting in the increased concentration of CH_3_ covalently bound to TED, which explains the experimentally observed higher amount of CH_3_I chemisorption (Supplementary Table 8).

## Discussion

Incorporating tertiary amine molecules within MIL-101-Cr gives rise to function-adjustable ROI molecular traps with record-high uptake capacities for ROIs capture from nuclear waste. In addition, these molecular traps exhibit excellent recyclability, which is not available for any currently known industrial adsorbents. Coupled with its exceptionally high chemical and thermal stability, high adsorption efficiency at a wide temperature range, and low cost, such molecular traps offer significant promise for ROIs capture from nuclear waste. We anticipate that such crystalline porous materials will become a new platform for effective capture of ROIs and that new molecular traps with optimized frameworks and improved performance will be developed in the near future.

## Methods

### Materials and measurements

Commercially available reagents were purchased in high purity and used without further purification. PXRD data were collected on a Rigaku Ultima-IV diffractometer or a Bruker AXS D8 Advance A25 Powder X-ray diffractometer. N_2_ gas sorption experiments were carried out on a Micromeritics 3Flex volumetric adsorption analyzer. Elemental analyses were performed on a Perkin-Elmer 2400 element analyzer. TGA was analyzed by a Q5000 thermogravimetric analyzer. The in situ infrared spectroscopic data were obtained using a Nicolet 6700 FTIR spectrometer (purchased from Thermo Scientific Inc, USA) equipped with a liquid N_2_-cooled mercury cadmium telluride MCT-A detector. A vacuum cell, purchased from Specac Ltd, UK (product number P/N 5850c), was placed in the sample compartment of the infrared spectrometer with the sample at the focal point of the beam (Supplementary Fig. 35). The MOFs (powder, ~2 mg) were gently pressed onto a KBr pellet (~1.3 cm diameter, 1–2 mm thick) and placed in the cell. The samples were first activated under atmospheric N_2_ flow at 150 °C for 3 h and then evacuated (base pressure <20 mtorr) for CH_3_I vapor exposure measurement. The infrared data were recorded during the vapor exposure. XPS measurements were performed on an ESCALAB 250 X-ray photoelectron spectroscopy, using Mg Kα X-ray as the excitation source. UV-Vis data were collected using a Shimadzu UV-3600 spectrophotometer. HRTEM-EDS analysis was performed in a FEI Tecnai G2 S-Twin with a field emission gun operating at 200 kV. Images were acquired digitally on a Gatan multiple CCD camera. The FEI Tecnai G2S-Twin is equipped with an EDS detector, which was used for elemental analysis of the nanocrystal composition. The ^1^H NMR data were collected on a Bruker AVANCE IIIHD console with 1.9 mm MAS probe. ICP was performed on a Perkin-Elmer Elan DRC II quadrupole inductively coupled plasma mass spectrometer (ICP-MS) analyzer. CH_3_I adsorption experiments were carried out on a homemade gravimetric adsorption analyzer modified from a thermogravimetric analyzer Q50 (TA Instruments).

### Synthesis of MIL-101-Cr

MIL-101-Cr was synthesized according to the reported procedure with minor modifications^61^. Typically, a solution containing chromium(III) nitrate Cr(NO_3_)_3_·9H_2_O (800 mg, 2.0 mmol), HNO_3_ (2.0 mmol), benzene-1,4-dicarboxylic acid (328 mg, 2.0 mmol), and 10 mL H_2_O was transferred to the PTFE/Teflon liner in a hydrothermal autoclave, which is heated at 210 °C for 8 h and cooled afterwards slowly to room temperature. The solid product was isolated as a green powder by centrifugation and washed three times with DMF, water, ethanol for 12 h at 80 °C, respectively. The final product was dried under vacuum at 150 °C for 24 h.

### Synthesis of MIL-101-Cr-TED

A resealable heavy-wall pressure flask was charged with MIL-101-Cr (1.0 g), TED (1.5 g), and benzene (50 mL). The flask was sealed and heated to 110 °C for 3 days. The green solid of MIL-101-Cr-TED was collected after washing the product with dry benzene, and then drying under vacuum at 200 °C for 3 h. Caution: the reaction was performed under high pressure with potential hazards. Elemental analysis: calculated: C: 47.63%; H: 3.97%, N: 6.17%; experimental: C: 47.58%; H: 4.14%; N: 6.38%. ICP: calculated: Cr 17.20%; experimental: Cr: 16.89%.

### Synthesis of MIL-101-Cr-HMTA

A similar procedure was used to synthesize MIL-101-Cr-HMTA. MIL-101-Cr (1.0 g), HMTA (1.5 g), and chloroform (50 mL) were loaded in a resealable flask. The flask was sealed and heated to 110 °C for 3 days. The product was collected, washed with dry chloroform, and then dried under vacuum at 200 °C for 3 h to yield the green solid, MIL-101-Cr-HMTA. Caution: the reaction was performed under high pressure with potential hazards. Elemental analysis: calculated: C: 44.86%; H: 3.74%, N: 11.63%; experimental: C: 44.87%; H: 4.08%; N: 12.15%. ICP: calculated: Cr: 16.20%; experimental: Cr: 15.87%.

### Synthesis of benchmark materials

The detail of synthesizing benchmark materials can be found in Supplementary Methods.

### Organic iodide and I_2_ adsorption measurements

CH_3_I adsorption experiments were carried out on a homemade gravimetric adsorption analyzer modified from a thermogravimetric analyzer Q-50 (TA Instruments). In a typical adsorption experiment, ~20 mg adsorbent sample was loaded on the thermobalance and activated at 200 °C for 2 h under N_2_ flow to ensure complete removal of residue solvents in the sample. The temperature was then reduced to either 30 or 150 °C and the gas flow was switched from pure N_2_ to a combination of a pure N_2_ gas stream and another N_2_ gas stream passing through a CH_3_I bubbler. The flow rates of the two gas streams were controlled via two gas flow controllers to achieve certain partial pressures. The adsorption amount was monitored by recording the sample weight. The gas flow was switched back to pure N_2_ after a plateau was reached. The sorption data of CH_3_CH_2_I and CH_3_CH_2_CH_2_I were collected under the same experimental procedure at 150 °C with a partial pressure of 0.1 and 0.05 atm, respectively. I_2_ adsorption isotherms were also obtained following a similar procedure at 150 °C with the I_2_ concentration at 150 ppm.

### Breakthrough experiment with or without humidity

The breakthrough experiment was conducted using a lab-scale fixed-bed reactor at 150 °C (Supplementary Fig. 36). In a typical experiment, the powder was activated at 150 °C for 3 h. Then 1.0 g of material was packed into a quartz column (5.8 mm I.D. × 150 mm) with silane-treated glass wool filling the void space. A helium flow (5 cm^3^ min^−1^) was used to purge the adsorbent. The flow of He was then turned off while dry N_2_ at a rate of 5 mL min^−1^ bubbled through CH_3_I and was allowed to flow into the column. The flow rate of CH_3_I was 8.872 mg min^−1^ (1.4 cm^3^ min^−1^), determined through trial and error. The effluent from the column was monitored using an online mass spectrometer (MS). Experiments in the presence of humidity were performed by injecting water into the gas mixture at a rate of 0.12 μL min^−1^ using a Fusion 100 syringe pump.

The absolute adsorbed amount of gas *i*(*q*_*i*_) is calculated from the breakthrough curve by the equation:

1 $${q_{\rm{i}}} = \frac{{{F_{\rm{i}}} \times {t_0} - {V_{{\rm{dead}}}} - \mathop {\int}\limits_0^{{t_0}} {{F_{\rm{e}}}\Delta t} }}{m}$$

where *F*_i_ is the influent flow rate of the specific gas (cm^3^ min^−1^); *t*_0_ is the adsorption time (min); *V*_dead_ is the dead volume of the system (cm^3^); *F*_e_ is the effluent flow rate of the specific gas (cm^3 ^min^−1^); and *m* is the mass of the sorbent (g).

It should be mentioned that the loading amounts obtained from the breakthrough experiments are slightly different from those from adsorption isotherm measurements. This discrepancy is mainly due to small errors associated with factors such as sample particle size, packing length, and packing density.

### Decontamination factor measurements

Decontamination factors (DFs) were measured using a similar system as breakthrough experiment. The concentration of CH_3_I was 50 ppm with or without I_2_ (150 ppm). 5 M nitric acid solution was introduced into a bubbler to allow the N_2_ (12 mL min^−1^) to carry the nitric acid and moisture vapors into the system, and the RH was 95% (23 °C). Heated lines temperature was kept at 150 °C to get in situ formation of NO_x_ during heating. The residual CH_3_I and I_2_ were collected at interval time by NaOH bubbler and tested by ICP-MS. The DFs were calculated based on the ICP-MS data.

### Ab initio calculations

Our ab initio calculations were performed at the density functional theory (DFT) level as implemented in the VASP^62, 63^ software, using the vdW-DF exchange-correlation function^64, 65^. We used PAW potentials with energy cutoff values of 600 eV and energies were converged to within 1×10^−4^ eV. Transition states were found with a common transition-state search algorithm.

### Ion-exchange experiments

A 10 mL aqueous solution of Orange G (0.073 mM) was added to a 20 mL vial, which was followed by addition of 30.0 mg of the respective MOF sample to form slurry at room temperature. During the stirring period, a small amount of mixture was drawn (~0.5 mL) and filtered immediately at selected time intervals through a 0.45 micron membrane filter, and the filtrates were analyzed using UV-vis spectroscopy to determine the concentration of the Orange G ions.

### Data availability

The data that support the findings of this study are available from the corresponding author on reasonable request.

## Electronic supplementary material

Supplementary Information
