# Supplementary material for: Capture of organic iodides from nuclear waste by metal-organic framework-based molecular traps
Source: Nat Commun. 2017 Sep 7;8:485. doi: 10.1038/s41467-017-00526-3 (PMC5589857; doi:10.1038/s41467-017-00526-3)
Supplement: Supplementary file 1 — Supplementary Information [file 41467_2017_526_MOESM1_ESM.pdf]

Description of Supplementary Files

File Name: Supplementary Information
Description: Supplementary Figures, Supplementary Tables, Supplementary Notes, Supplementary Methods and Supplementary References

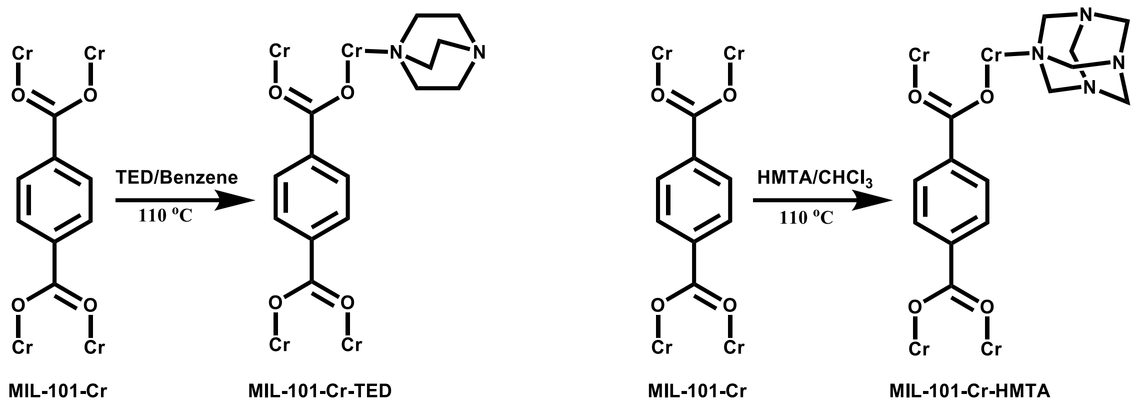

Supplementary Figure 1 | Synthetic route to MIL-101-Cr-TED (left) and MIL-101-Cr-HMTA (right).

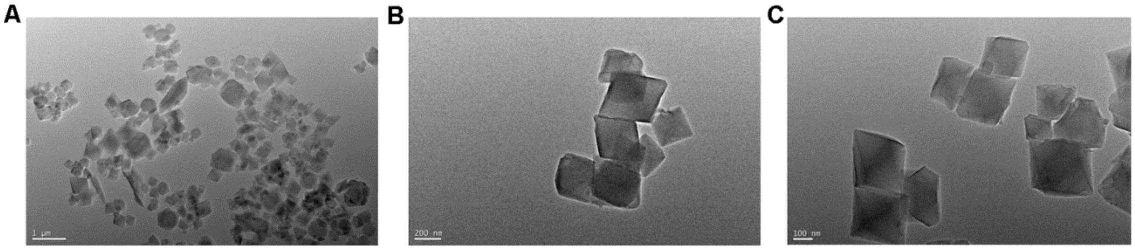

Supplementary Figure 2 | The TEM images. (A) MIL-101-Cr, (B) MIL-101-Cr-TED and (C) MIL-101-Cr-HMTA.

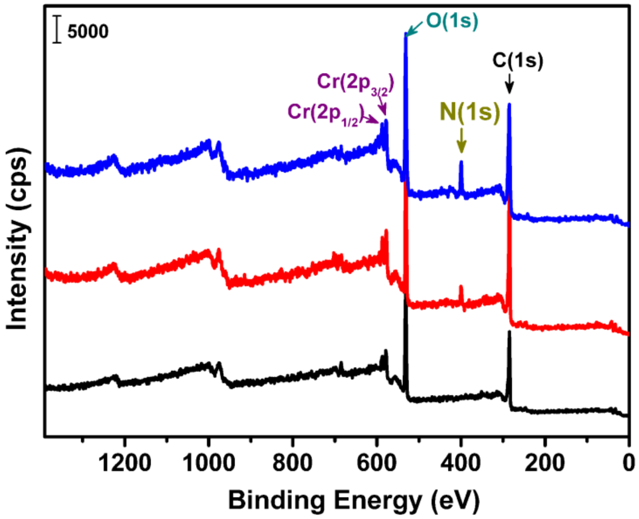

Supplementary Figure 3 | The XPS spectra of MIL-101-Cr (black), MIL-101-Cr-TED (red) and MIL-101-Cr-HMTA (blue).

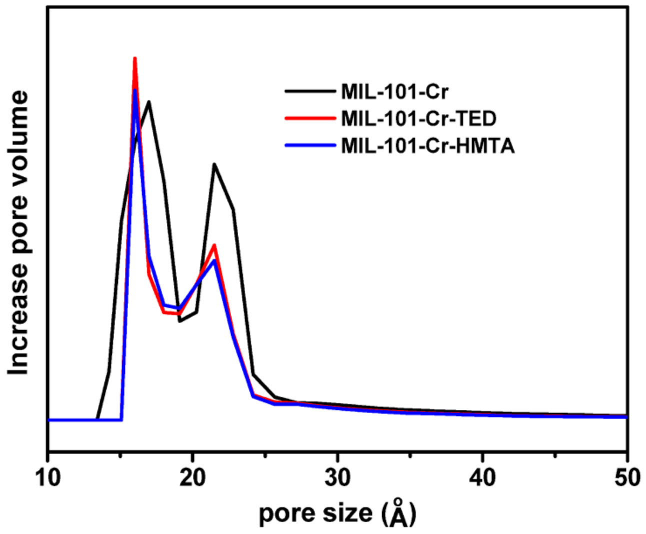

Supplementary Figure 4 | BJH pore size distribution calculated from nitrogen adsorption isotherms at 77 K.

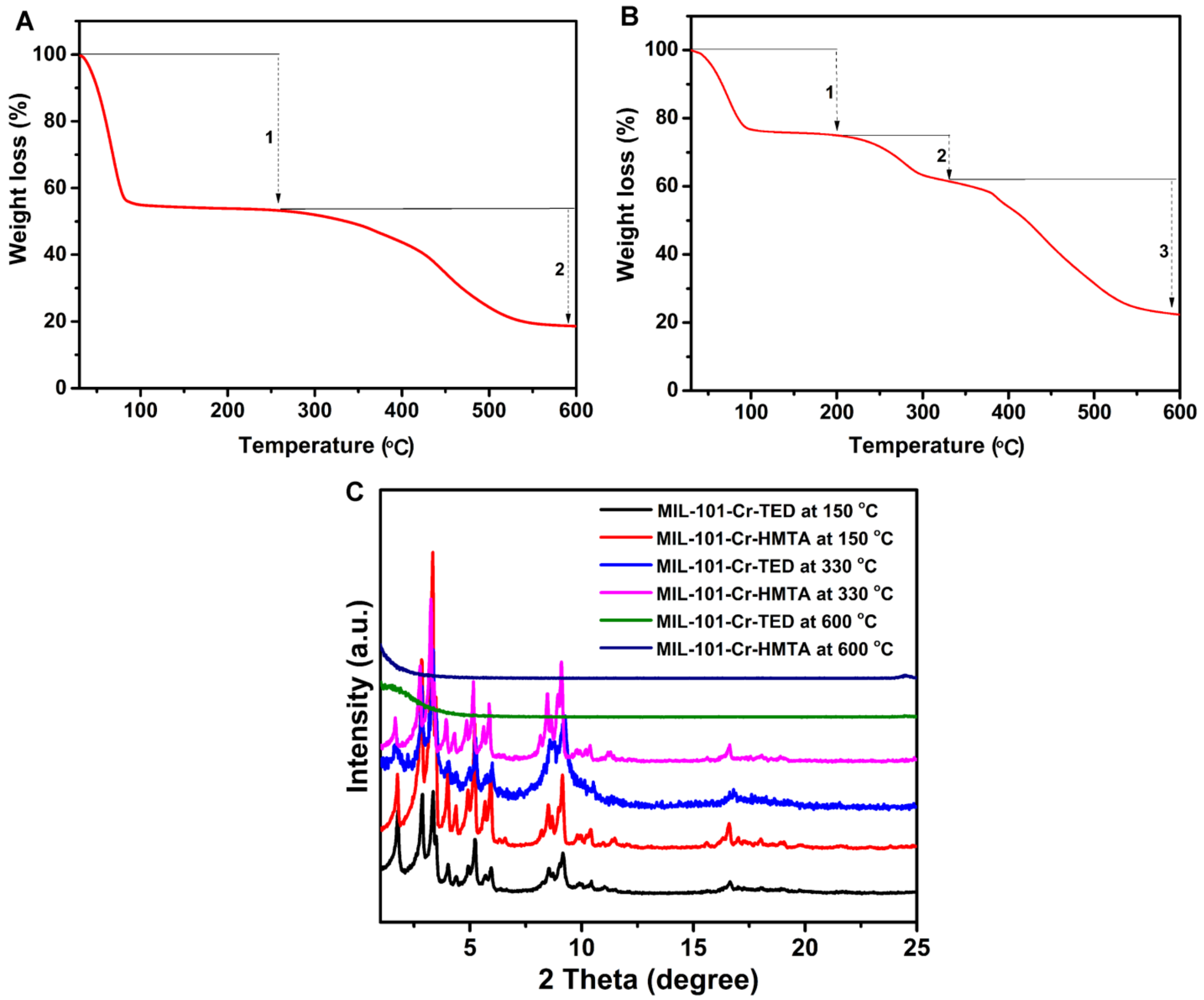

Supplementary Figure 5 | The TG profiles of (A) MIL-101-Cr-TED and (B) MIL-101-Cr-HMTA and (C) PXRD patterns of MIL-101-Cr-TED and MIL-101-Cr-HMTA after being heated at different temperatures.

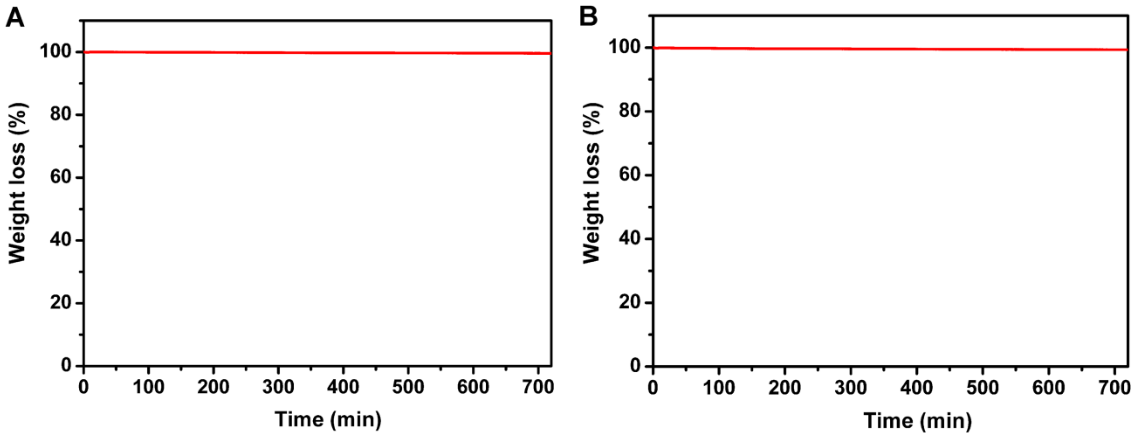

Supplementary Figure 6|The isothermal TG profiles of (A) MIL-101-Cr-TED and (B) MIL-101-Cr-HMTA at 150 °C for 12 h.

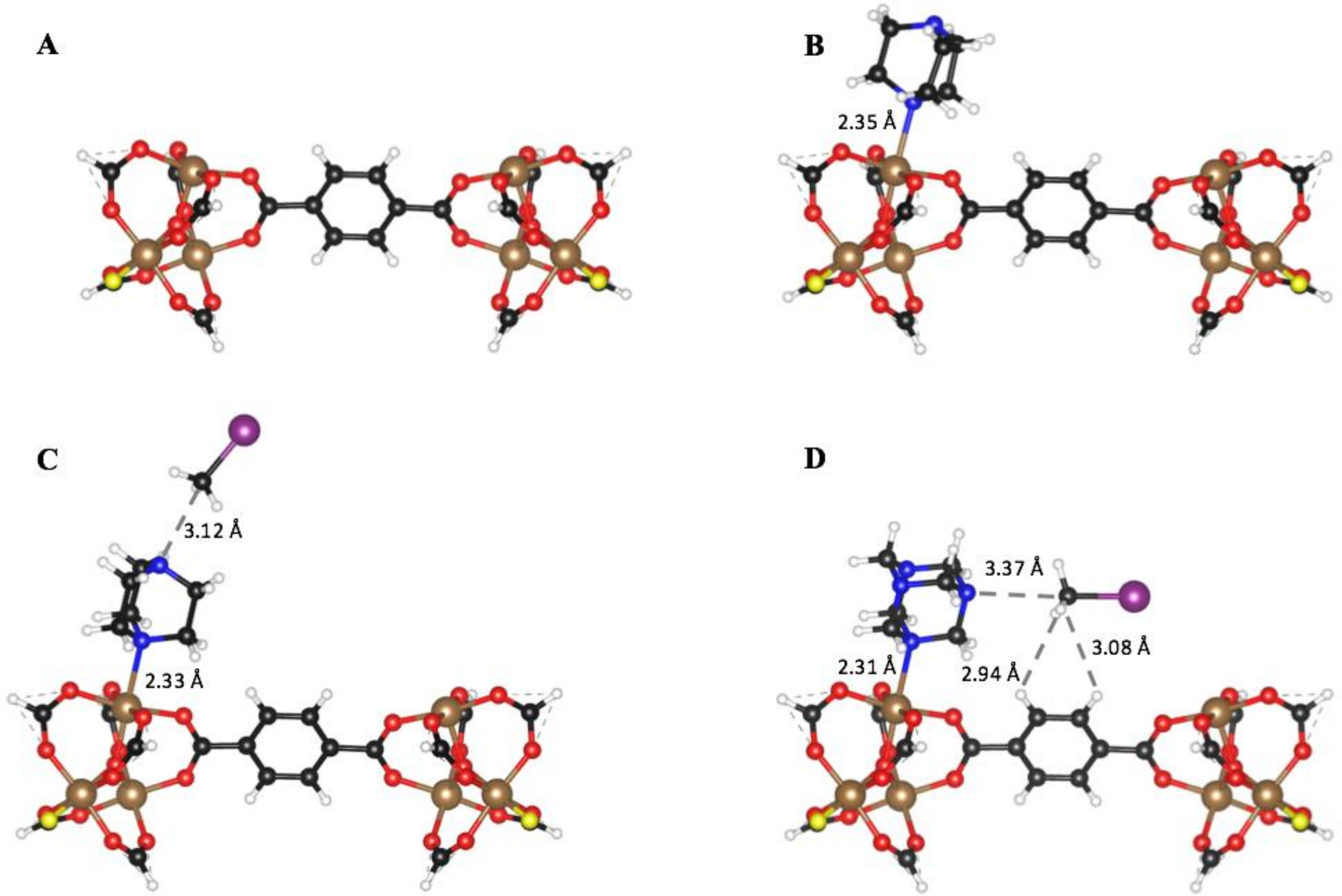

Supplementary Figure 7 | Models used for *ab initio* calculations include (A) the unit cell of the cut out of MIL-101-Cr, (B) TED bound to the MIL-101-Cr, (C) CH_3I bound to TED, which is bound to the MIL-101-Cr, and (D**)** CH_3I bound to HMTA, which is bound to the MIL-101-Cr cut out. The color coding is as follows: C: black; Cr: gold; F: yellow; H: white; N: blue; O: red; I: purple.

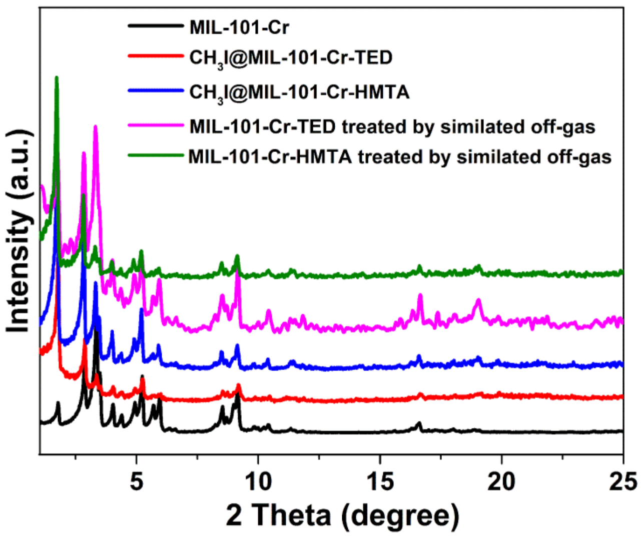

Supplementary Figure 8│The PXRD patterns of CH_3I loaded samples of MIL-101-Cr-TED and MIL-101-Cr-HMTA, and two samples after the capture of simulated off-gas mixture and the pristine MIL-101-Cr sample.

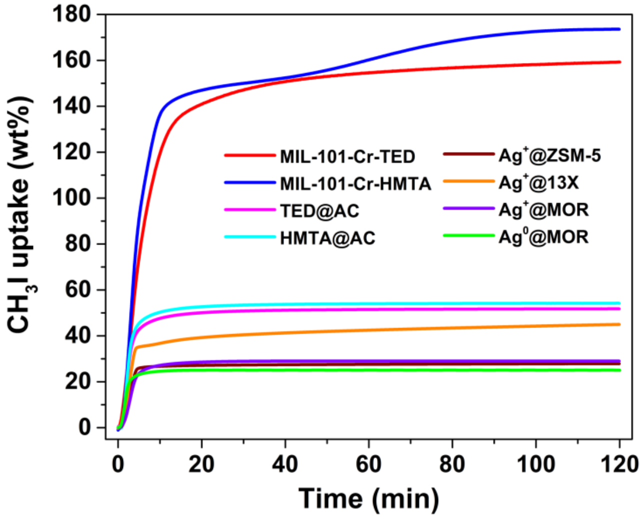

Supplementary Figure 9 | Adsorption isotherms of CH_3I in MIL-101-Cr-TED, MIL-101-Cr-HMTA and selected benchmark sorbent materials collected at 30 °C with a CH_3I partial pressure of 0.2 atm.

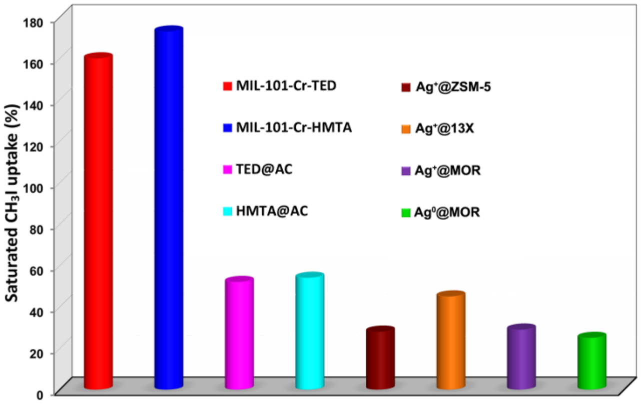

Supplementary Figure 10 | Saturated CH_3I uptake in MIL-101-Cr-TED, MIL-101-Cr-HMTA and selected benchmark sorbent materials at 30 °C with a partial pressure of 0.2 atm for CH_3I.

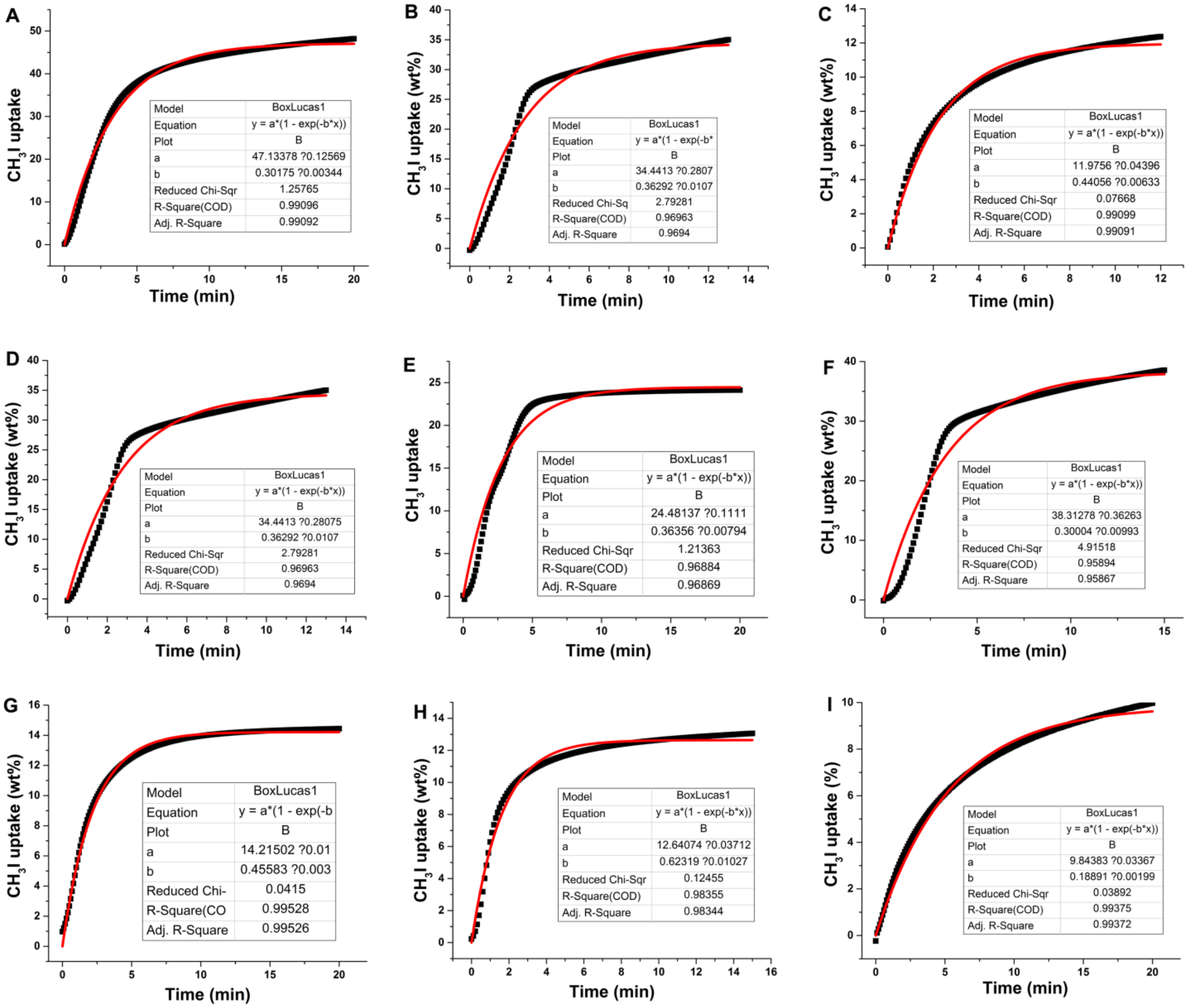

Supplementary Figure 11 | The experimental uptake (black) and kinetic fitting curve (red) of MIL-101-Cr-TED, MIL-101-Cr-HMTA, TED@AC, HMTA@AC, Ag^+@ZSM-5, Ag^+@13X, Ag^+@MOR, Ag^0@MOR, and MIL-101-Cr.

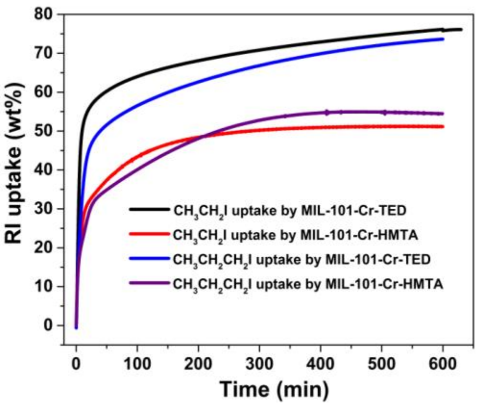

Supplementary Figure 12 | The adsorption isotherms of CH_3CH_2I and $CH_3CH_2CH_2I$ in MIL-101-Cr-TED and MIL-101-Cr-HMTA at 150 °C.

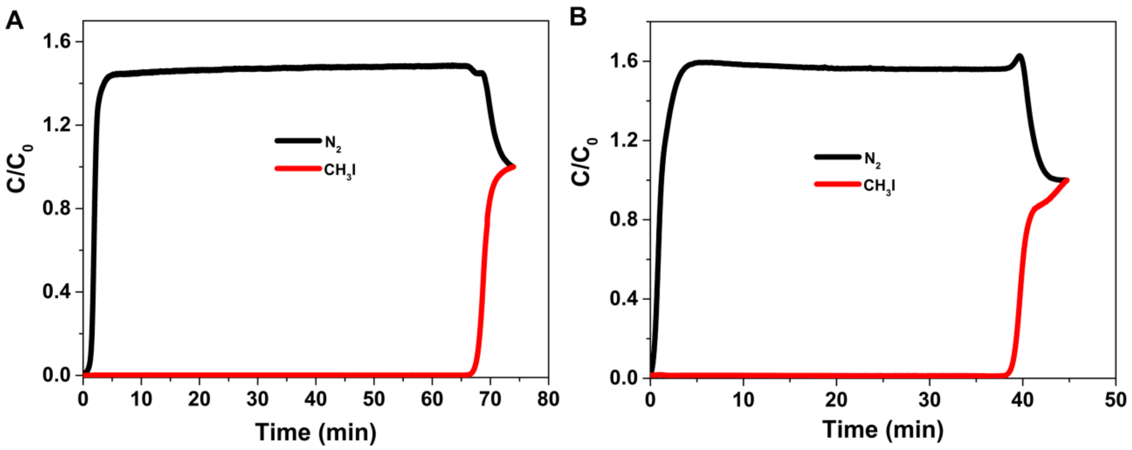

Supplementary Figure 13 | The breakthrough curves of CH_3I for MIL-101-Cr-TED at 150 °C under (A) dry and (B) humidity (RH = 81%, 23 °C) conditions.

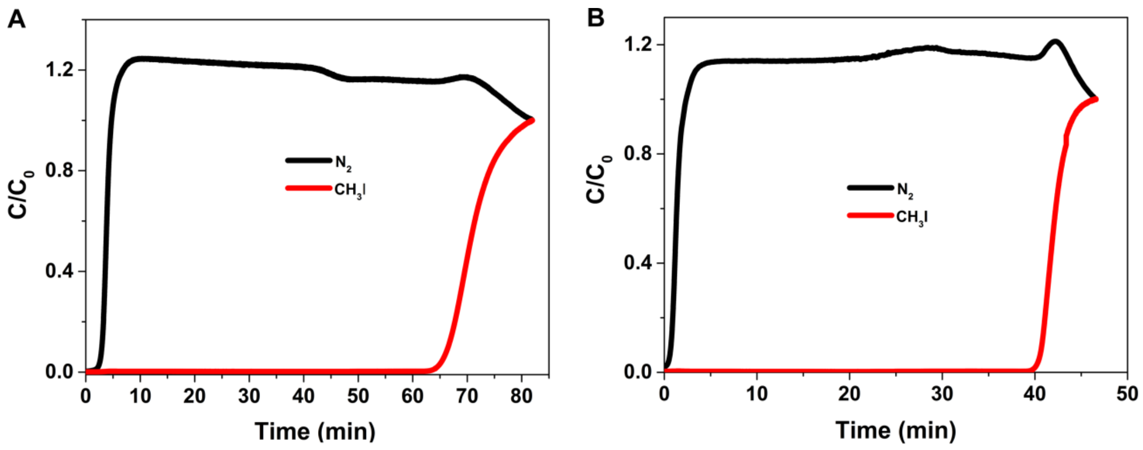

Supplementary Figure 14 | The breakthrough curves of CH_3I for MIL-101-Cr-HMTA at 150 °C under (A) dry and (B) humidity (RH = 81%, 23 °C) conditions.

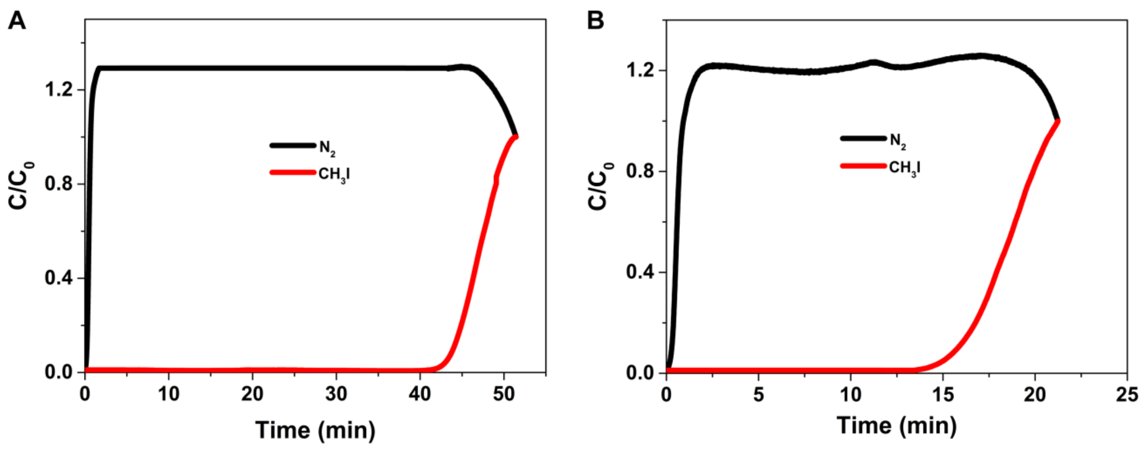

Supplementary Figure 15 | The breakthrough curves of CH_3I for Ag^+@13X at 150 °C under (A) dry and (B) humidity (RH = 81%, 23 °C) conditions.

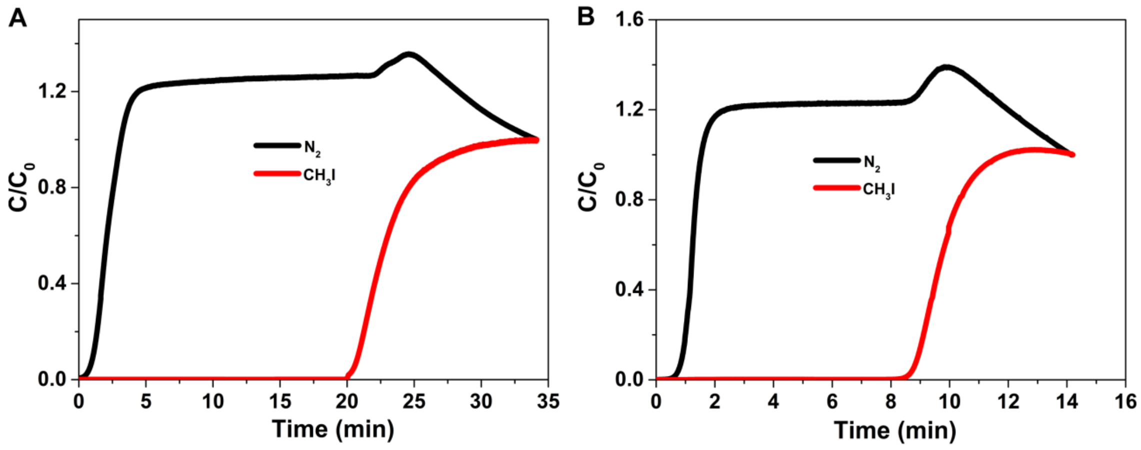

Supplementary Figure 16 | The breakthrough curves of CH_3I for Ag^0@MOR at 150 °C under (A) dry and (B) humidity (RH = 81%, 23 °C) conditions.

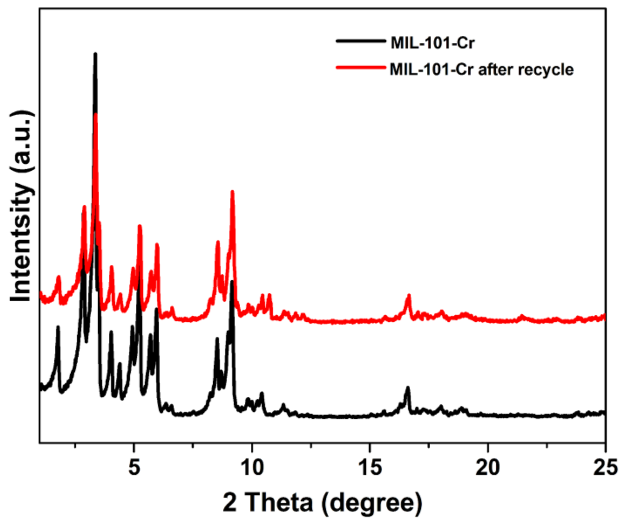

Supplementary Figure 17│The PXRD patterns of MIL-101-Cr and MIL-101-Cr after recycle experiments.

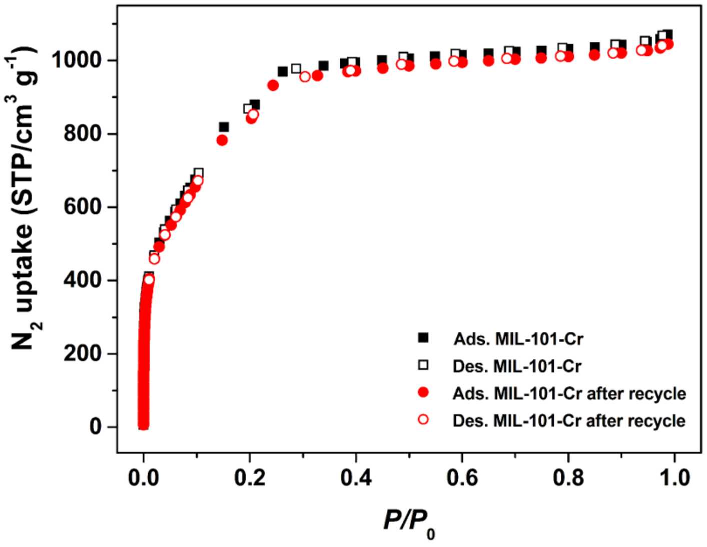

Supplementary Figure 18 | The 77K N_2 sorption isotherms of MIL-101-Cr and MIL-101-Cr after recycle experiments.

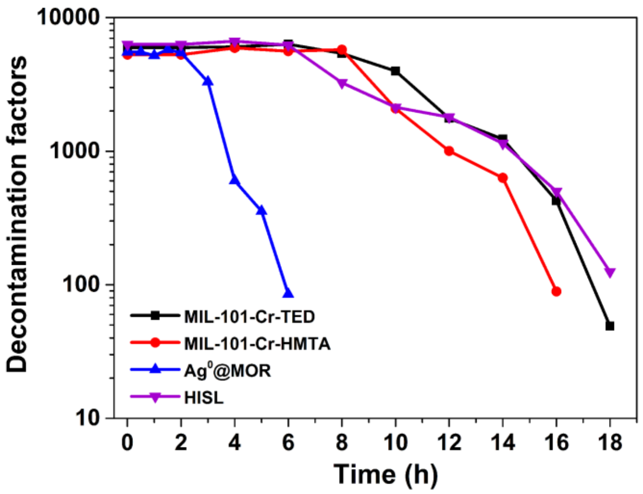

Supplementary Figure 19│Decontamination factors obtained from room temperature breakthrough experiments of MIL-101-Cr-TED, MIL-101-Cr-HMTA and selected benchmark materials under simulated gas mixture conditions.

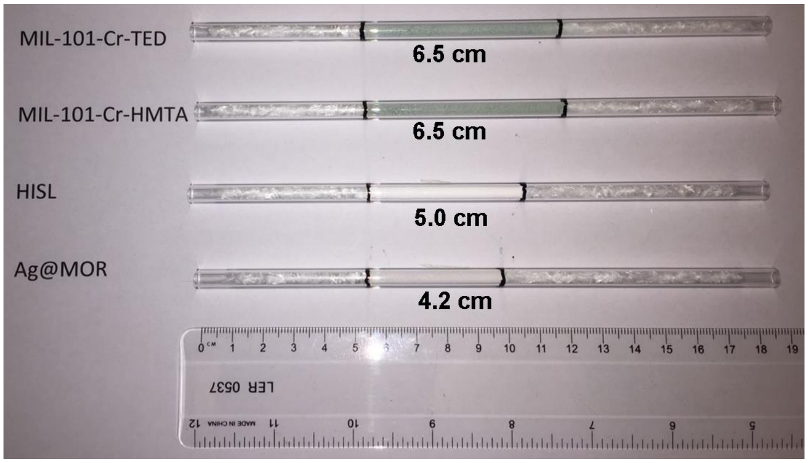

Supplementary Figure 20│Calculation of packing densities of selected samples. 300.0 mg activated samples were packed into a column with inner diameter of 0.36 cm, the packing lengths are shown in the picture. The calculated packing density: 0.46 g cm^{-3} for MIL-101-Cr-TED, 0.46 g cm^{-3} for MIL-101-Cr-HMTA, 0.59 g cm^{-3} for HISL, and 0.70 g cm^{-3} Ag^{0}@MOR.

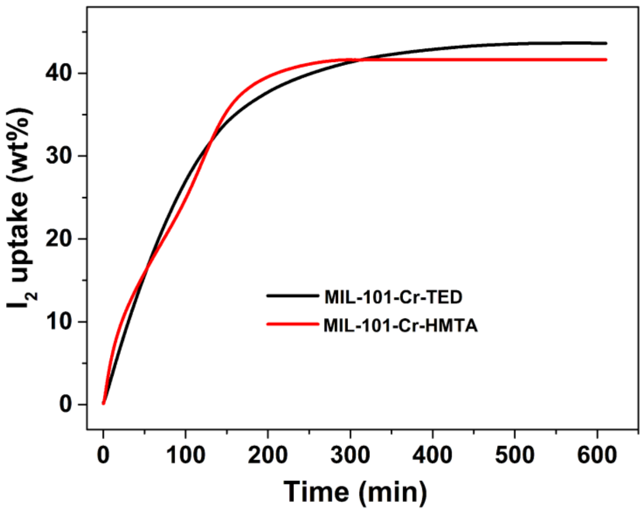

Supplementary Figure 21 | The I_2 adsorption isotherms in MIL-101-Cr-TED and MIL-101-Cr-HMTA at 150 °C and 150 ppm I_2 concentration.

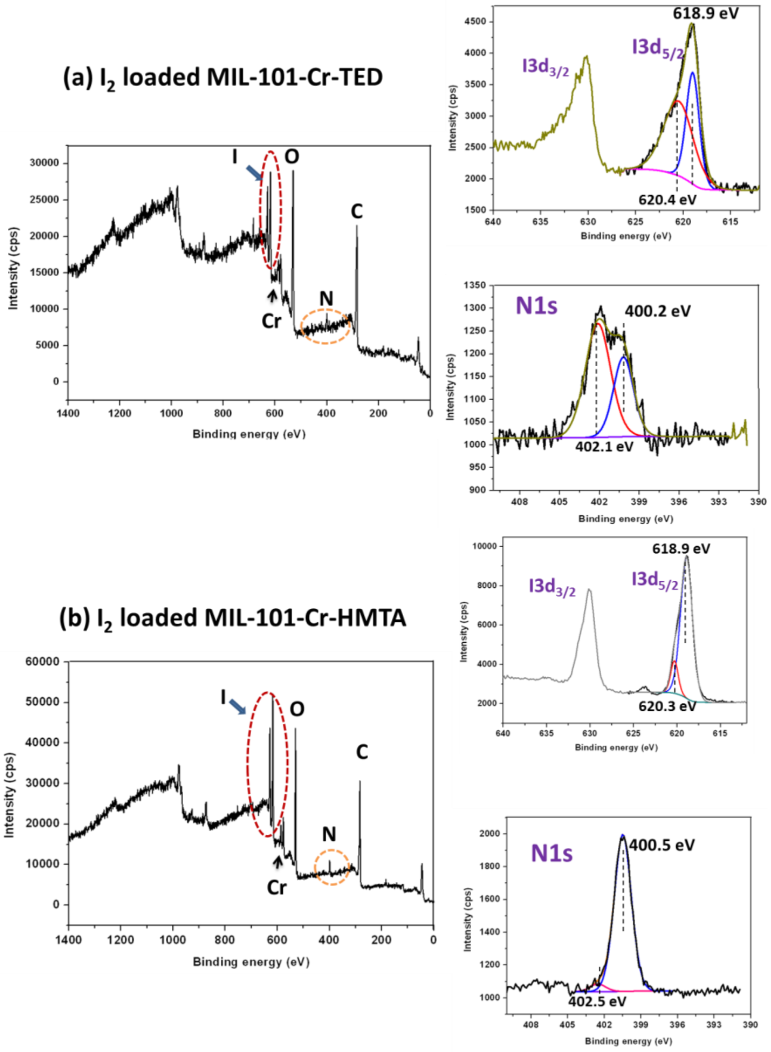

Supplementary Figure 22 | XPS spectra of I_2 loaded (a) MIL-101-Cr-TED and (b) MIL-101-Cr-HMTA samples. The samples of MIL-101-Cr-TED and MIL-101-Cr-HMTA were exposed to evaporated I_2 at 150 °C for 30 min and transferred to XPS chamber for the measurements. The XPS data clearly show that I_2 is adsorbed inside both MOFs, dominantly as the $I3d_{5/2}$ and $I3d_{3/2}$ peaks. $I3d_{5/2}$ peak can be readily deconvoluted into two components at 618.9 eV and 620.4 eV in different ratio. The former is attributed to triiodide (I_3^-) and the latter is due to pentaiodide (I_5^-).[1]

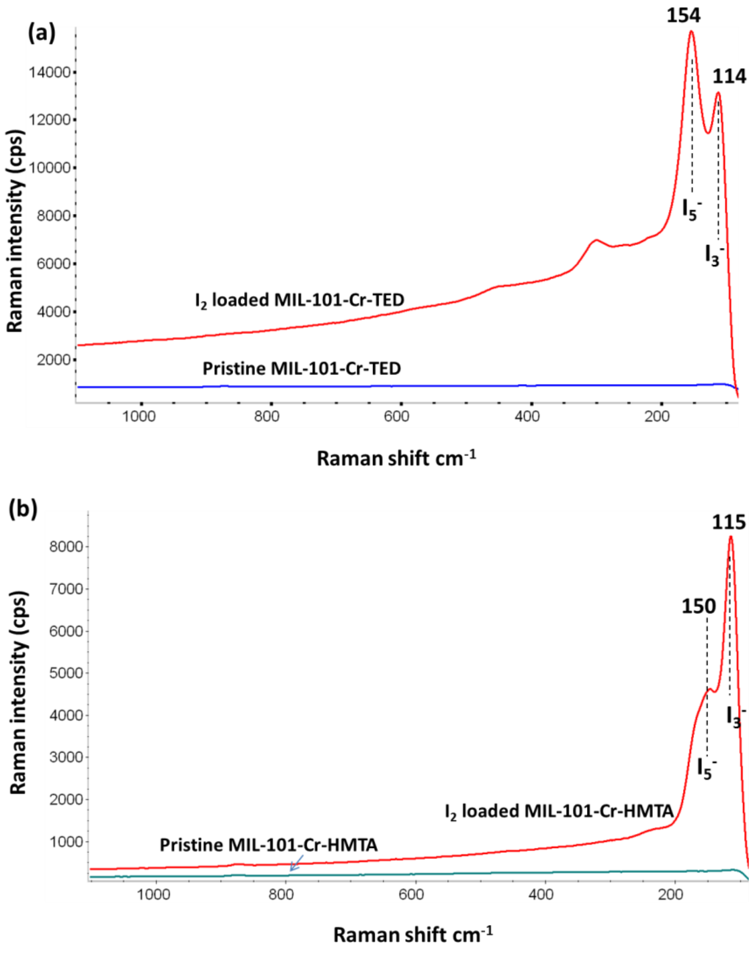

Supplementary Figure 23│Raman spectra of I_2 loaded (a) MIL-101-Cr-TED and (b) MIL-101-Cr-HMTA samples. The presence of I_3^- band at 154, 149 cm^{-1} for MIL-101-Cr-TED and I_5^- band at 114, 115 cm^{-1} for MIL-101-Cr-HMTA can be observed in the Raman spectra.[2]

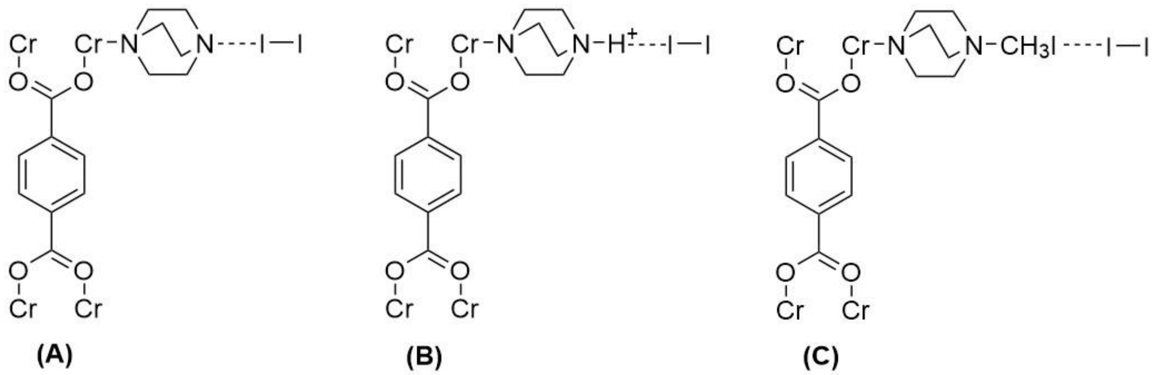

Supplementary Figure 24│The possible binding modes of I_2 to MIL-101-Cr-TED under the simulated gas-mixture conditions.

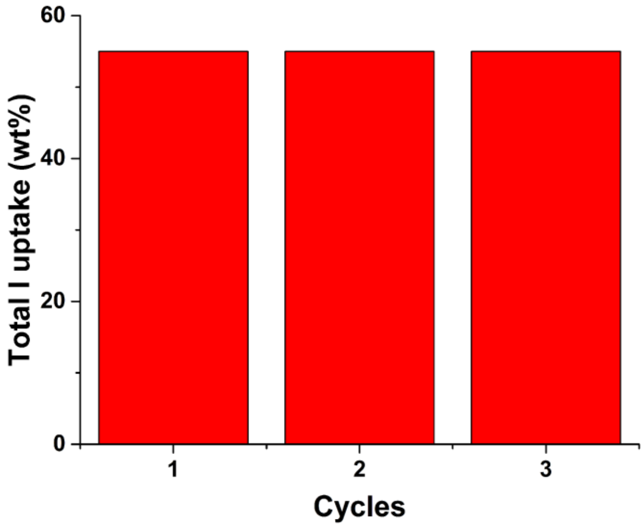

Supplementary Figure 25|The recyclability test results of MIL-101-Cr-TED under simulated gas mixture conditions. The MIL-101-Cr was recovered by washing iodine loaded samples with HCl (3 M), DMF, and ethanol twice. The sample was then collected, activated, and re-functionalization to form MIL-101-Cr-TED for the next cycle.

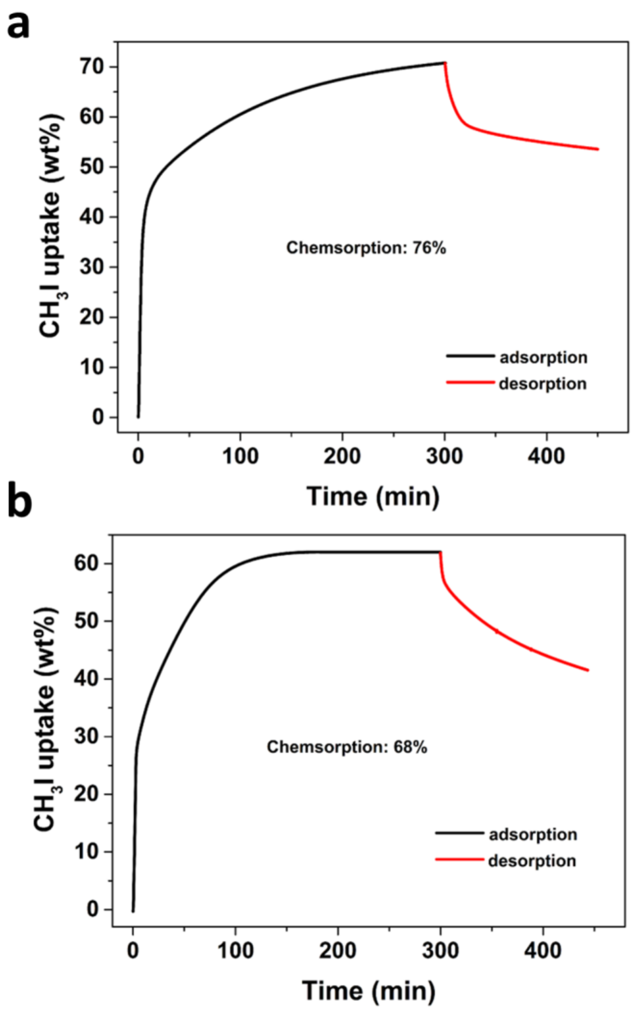

Supplementary Figure 26 | The adsorption and desorption curves of CH_3I for (a) MIL-101-Cr-TED and (b) MIL-101-Cr-HMTA at 150 °C. Desorption of CH_3I was performed at 150 °C under 90 mL/min N_2 stream.

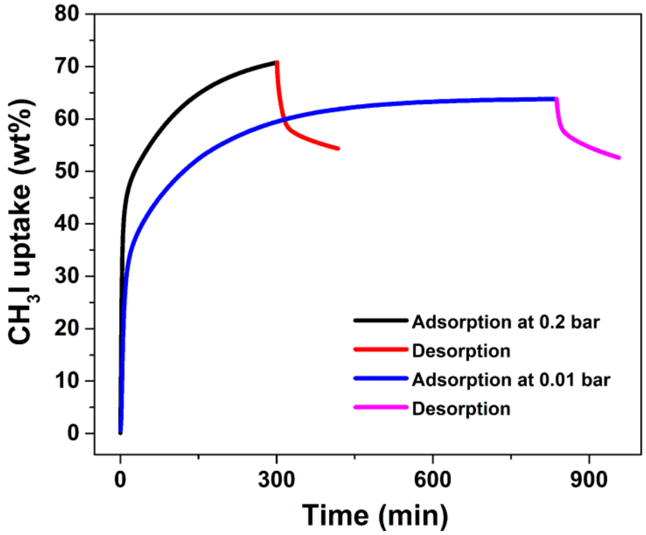

Supplementary Figure 27 | Adsorption-desorption isotherms of MIL-101-Cr-TED at 150 °C and different partial pressures. The desorption experiment was carried out at 150 °C and under N_2 stream for 120 min (90 mL/min). Essentially the same amount of chemisorption was achieved at different partial pressures (~54 and 53 wt% at 0.2 and 0.01 bar, respectively), suggesting very little dependence on pressure.

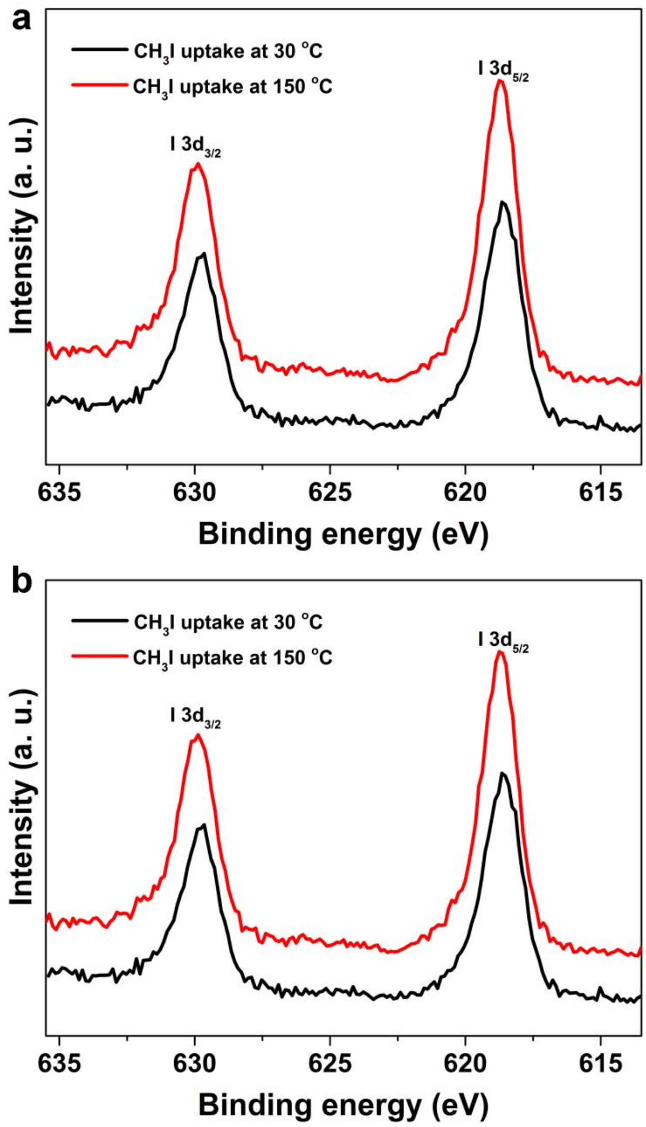

Supplementary Figure 28│I 3d XPS of (a) MIL-101-Cr-TED and (b) MIL-101-Cr-HMTA after CH_3I loading at 30 and 150 °C. All CH_3I loaded samples were heated at 150 °C for 3 h under N_2 stream prior to the XPS experiments.

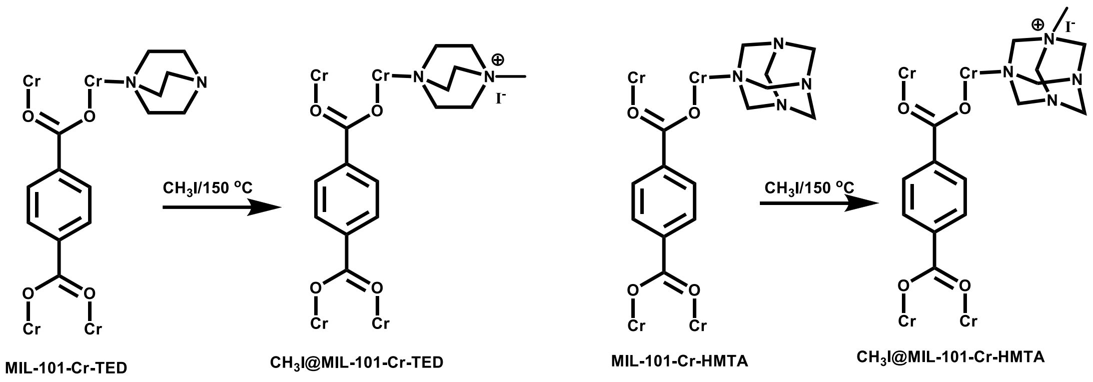

Supplementary Figure 29 | Proposed chemical sorption mechanism.

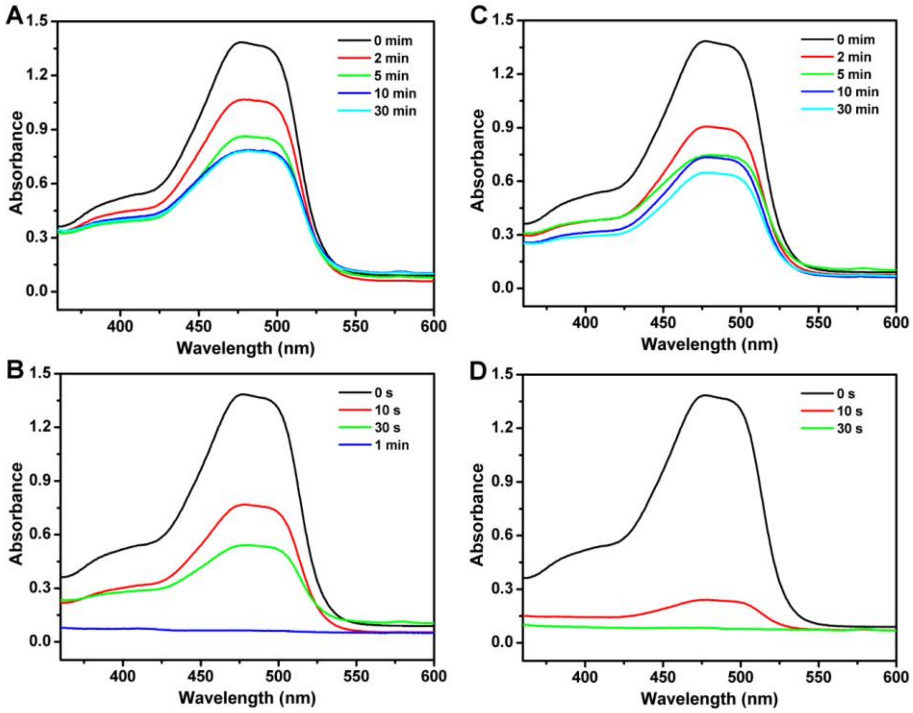

Supplementary Figure 30 | The UV-vis spectra of an aqueous solution of Orange G dye in the presence of (A) MIL-101-Cr-TED, (B) CH_3I loaded MIL-101-Cr-TED, (C) MIL-101-Cr-HMTA, and (D) CH_3I loaded MIL-101-Cr-HMTA monitored with time. Note: Each time interval given in the Figure represents the point when a small sample was drawn out.

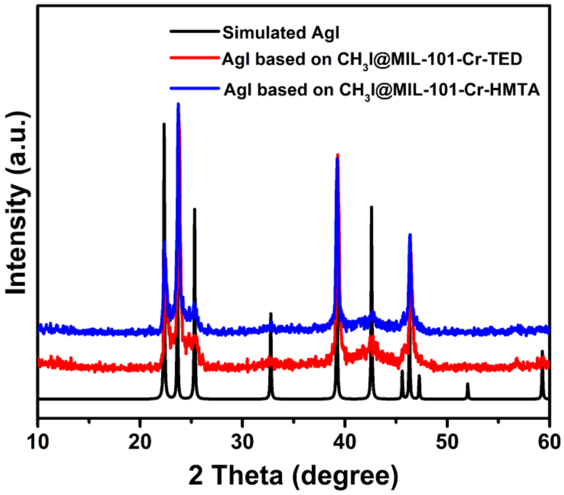

Supplementary Figure 31 | The PXRD pattern of AgI formed after $AgNO_3$ titration of dye exchanged filtrates for MIL-101-Cr-TED and MIL-101-Cr-HMTA.

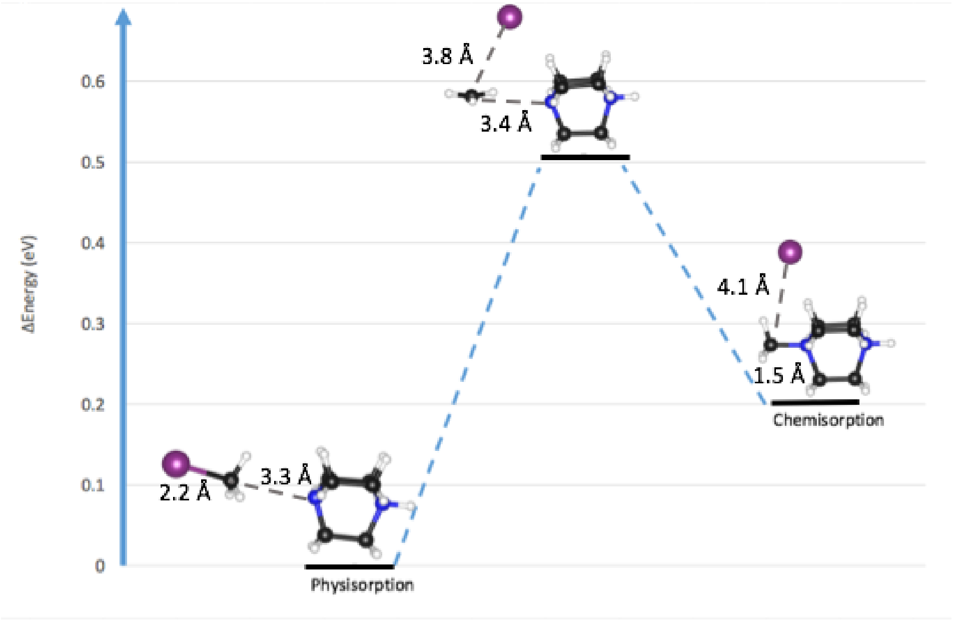

Supplementary Figure 32│Diagram of transition state calculation of CH_3I bound to capped TED. The reactant involves van der Waals interactions of CH_3I and TED, the transition state involves the broken CH_3 and I bond, and the product is CH_3 bound to the N of TED with I^- dissociated from the molecule. The color coding is as follows: C: black; H: white, N: blue, I: purple.

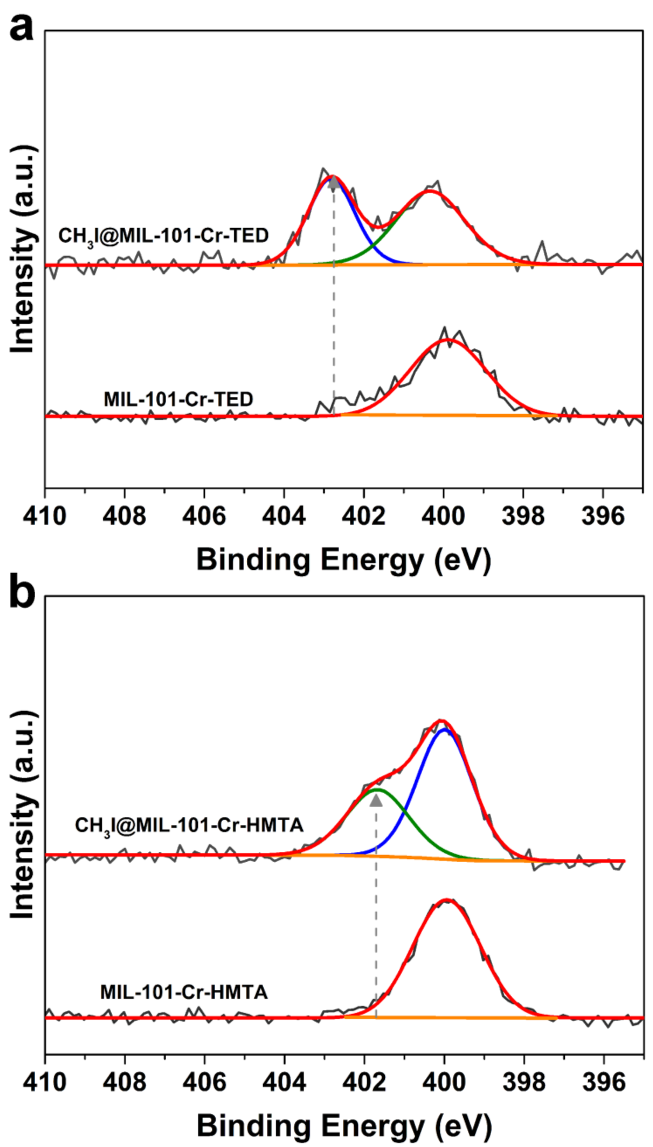

Supplementary Figure 33 | N 1s XPS spectra of (a) MIL-101-Cr-TED and CH_3I@MIL-101-Cr-TED (30 °C), and (b) MIL-101-Cr-HMTA and CH_3I@MIL-101-Cr-HMTA (30 °C).

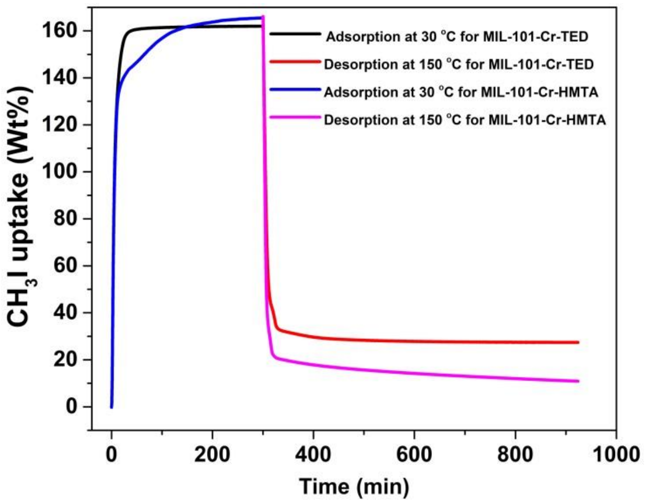

Supplementary Figure 34│ The adsorption-desorption isotherms of CH_3I for MIL-101-Cr-TED and MIL-101-Cr-HMTA at 30 °C. Desorption of CH_3I was performed at 150 °C under 90 mL/min N_2 stream.

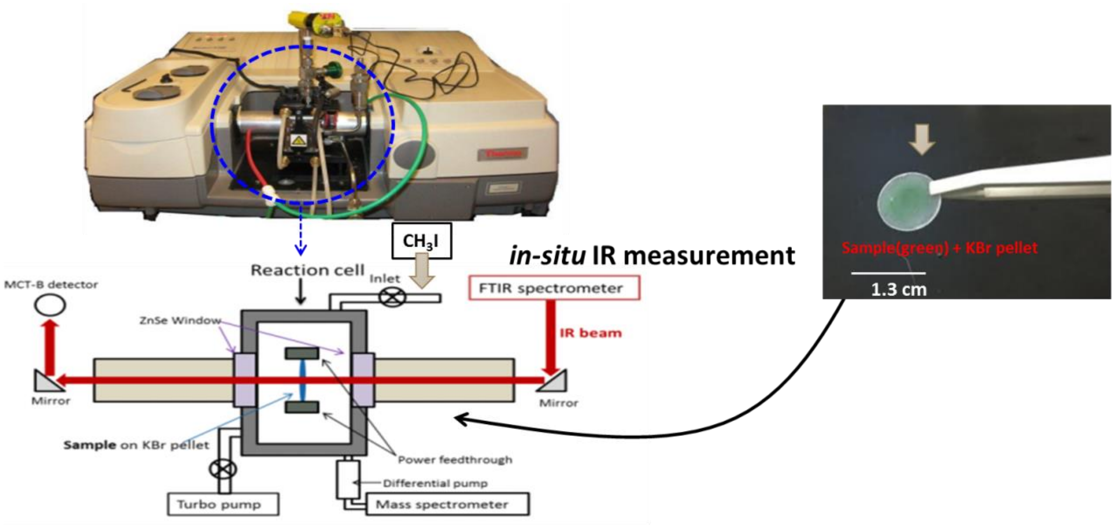

Supplementary Figure 35 | Representation of the in-situ IR experimental apparatus.

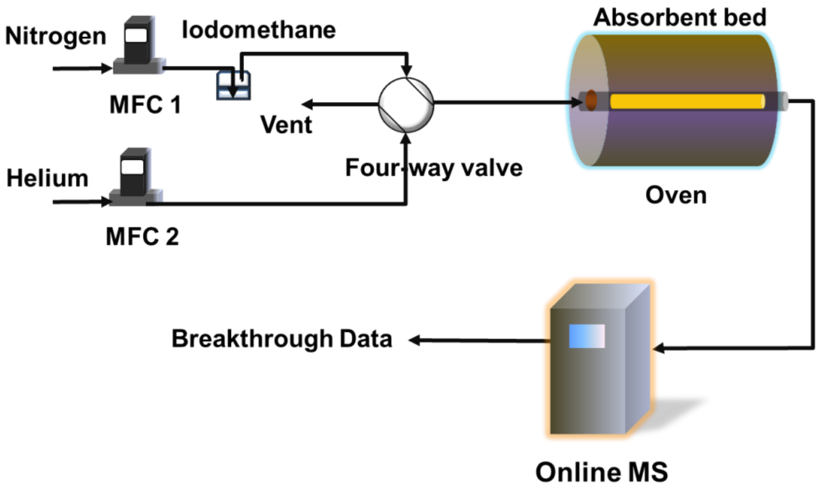

Supplementary Figure 36 | Representation of the breakthrough experimental apparatus.

Supplementary Table 1 | Binding energy of selected molecules to the OMSs of the MIL-101-Cr structure.

Molecules	E_b [eV]	Bond Length Cr–X [Å]
CH_3I	–0.1603	3.44
H_2O	–0.7356	2.22
TED	–1.1042	2.35
HMTA	–1.337	2.31

Supplementary Table 2 | The kinetic constants of MIL-101-Cr-TED, MIL-101-Cr-HMTA and other comparable adsorbent materials.

Sample	k_1	Sample	k_1	Sample	k_1
MIL-101-Cr-TED	0.30	HMTA@AC	0.27	Ag^{+}@MOR	0.46
MIL-101-Cr-HMTA	0.36	Ag@ZSM-5	0.36	Ag^{0}@MOR	0.62
TED@AC	0.44	Ag@13X	0.30	MIL-101-Cr	0.19

**Supplementary Table 3 | ** The compared performance of saturated CH_3I uptake in MIL-101-Cr-TED, MIL-101-Cr-HMTA, and Ag@MOR under the dry and humidity conditions.

**Material**	**MIL-101-Cr-TED**	**MIL-101-Cr-HMTA**	**Ag@MOR**
CH_3I uptake at 150 ^oC (dry) (wt%)	61.1	62.8	21.2
CH_3I uptake at 150 ^oC (humid) (wt%)	35.2	37.2	7.7
CH_3I uptake at 150 ^oC (dry) (g cm^{-3})[a]	0.28[a] (0.35[b])	0.29[a] (0.39[b])	0.15[a] (0.47[b])
CH_3I uptake at 150 ^oC (humid) (g cm^{-3})[a]	0.16[a] (0.20[b])	0.17[a] (0.23[b])	0.05[a] (0.17[b])

[a] Based on the packing density of samples given in Supplementary Figure 20; [b] Based on the crystal density of the samples. MIL-101-Cr-TED: 0.58 g cm^{-3}; MIL-101-Cr-HMTA: 0.62 g cm^{-3}; Ag@MOR: 2.23 g cm^{-3}; HISL: 1.81 g cm^{-3}.

**Supplementary Table 4 | **Comparison of performance and cost of MIL-101-Cr-TED and industrial adsorbent Ag^0@MOR.

Material	Stability[a]	Maximum uptake capacity at 150 °C (wt%)[b]	Decrease in uptake capacity under humidity[c]	Nuclear processing facilities' regulatory standards[d]	Full recyclability	Cost ($\$/g_{CH_3I}$)[e]
MIL-101-Cr-TED	Yes	71 wt%	42.4%	Yes	Yes	0.29
Ag^0@MOR	Yes	16 wt%	63.7%	Yes	No	10.2

[a] Including both acid stability and thermal stability; [b] based on adsorption isotherm data; [c] RH = 81%; [d] Nuclear processing facilities' regulatory standards require a DF of 3000 (99.967% of active species removed) for CH_3I reprocessing; [e] The estimated cost is based on the consumption of TED and Ag^0@MOR, respectively, for a single cycle. The cost of MIL-101-Cr is not included as it is fully recyclable. The uptake amount of CH_3I used for cost calculation is based on the data obtained under humid conditions. The prices used for cost calculations are from Sigma-Aldrich (TED and silver, 500 g/pack) and Alfa Aesar (MOR, 500 g/pack).

Supplementary Table 5 | The comparison of total iodine uptakes (I_2 and CH_3I) for MIL-101-Cr-TED, MIL-101-Cr-HMTA, Ag^0@MOR and pure silica zeolite HISL under the conditions of simulated gas mixtures including I_2 (150 ppm), CH_3I (50 ppm), humidity (RH = 95%), HNO_3 and NO_x at 150 °C and room temperature and 25 mg samples were used in the experiments.

Material	MIL-101-Cr-TED	MIL-101-Cr-HMTA	Ag^0@MOR	HISL
Total iodine uptake at 150 °C (wt%)	38	33	5	16
Total iodine uptake at RT (wt%)	55	44	8	44
Total iodine uptake at 150 °C (g cm^{-3})	0.18^a (0.22^b)	0.15^a (0.21^b)	0.04^a (0.11^b)	0.09^a (0.29^b)
Total iodine uptake at RT (g cm^{-3})	0.25^a (0.32^b)	0.20^a (0.27^b)	0.06^a (0.18^b)	0.26^a (0.79^b)

Note: The carrier gas flow rate: 12 mL min^{-1} for all materials except Ag^0@MOR with 6 mL min^{-1}.
[a] Based on the packing density of samples given in Supplementary Scheme 4, [b] Based on the crystal density of the samples. MIL-101-Cr-TED: 0.58 g cm^{-3}; MIL-101-Cr-HMTA: 0.62 g cm^{-3}; Ag@MOR: 2.23 g cm^{-3}; HISL: 1.81 g cm^{-3}.

Supplementary Table 6 | Binding energies for possible interaction modes of I_2 with MIL-101-Cr-TED.

mode	E_b [eV]
TED--I_2	–0.654
H^+--I_2	–1.667
CH_3I--I_2	–0.249

Supplementary Table 7 | Binding energy of CH_3I to the TED/HMTA molecules capped on the OMSs of MIL-101-Cr.

Capped MIL-101	E_b [eV]	Bond Length N–CH_3I [Å]
CH_3I–TED	–0.115	3.12
CH_3I–HMTA	–0.119	3.37

Supplementary Table 8 | The comparison of chemisorption of CH_3I in MIL-101-Cr-TED and MIL-101-Cr-HMTA at 30 and 150 °C at a CH_3I partial pressure of 0.2 atm.

Material	MIL-101-Cr-TED	MIL-101-Cr-HMTA
30 °C	27 wt%	12 wt%
150 °C	54 wt%	43 wt%

Supplementary Note 1

For MIL-101-Cr-TED, a two-step weight loss was observed. The first step (before 250 °C) is attributed to the loss of solvent, while the second step (above 250 °C) is most likely related to the loss of TED molecules followed by degradation of the framework above 330 °C. For MIL-101-Cr-HMTA, a three-step weight loss was observed. The first step before 200 °C is due to the solvent loss, and the second step before 330 °C may attribute to the thermal decomposition of HMTA molecules. The third step is mainly due to the degradation of the MIL-101-Cr framework.

Supplementary Note 2

(a) Computational details.

Ab initio modeling was performed at the density functional theory level using VASP[3,4] with the vdW-DF exchange-correlation functional.[5-8] Calculations were performed on a cut out of Cr MIL-101, as the full structure is computationally beyond *ab initio* analysis. Our MIL-101 model contained the commonly used 66-atom cluster shown in Supplementary Fig. 7.[9] PAW pseudopotentials were implemented with an energy cutoff of 600 eV and only the Γ point was used. The reaction barrier was calculated using a transition-state search algorithm, i.e., the climbing-image nudged-elastic-band (NEB) method.[10,11] This is a method for finding saddle points and minimum energy barriers between given start and end states. In our case, the start and end states were optimized according to the *ab initio* method mentioned above and we found them to be the configurations depicted in the insets in Supplementary Figure 7. NEB then works by optimizing a number of intermediate images along the pathway connecting the start and end states. Each image finds the lowest energy possible—while maintaining equal spacing to neighboring images—through a constrained optimization by adding spring forces along the band between images and projecting out the component of the force due to the potential perpendicular to the band.

All possible binding configurations were considered for each molecule. TED was tested on the open-metal site, the organic linker, and associating with the benzene ring of the organic linker; we found the Cr–N bond to be the strongest. We further found that the HMTA is taking on the same configuration as the TED molecule due to its similar structure. CH_3I was tested by binding both the methyl group and the iodine on the open-metal site and on the nitrogen of the TED molecule. We found that the methyl group provided the strongest interaction in both cases. The water is small enough that the optimal configuration can be obtained via simple structural relaxation, where the oxygen is bound to the metal. The Cr open-metal sites not occupied by fluorine are equivalent, as well as the N sites on TED/HMTA binding molecules, so only one open-metal site was tested when binding molecules.

(b) Binding energies of molecules on MIL-101-Cr.

Binding of four different molecules to the Cr atom of MIL-101-Cr was studied, with results shown in Supplementary Table 1. As mentioned earlier, all necessary configurations were tested and the lowest-energy configurations are reported. The CH_3I itself binds only weakly to the Cr through van der Waals interactions, while the TED and HMTA show strong, covalent binding. The binding energies also suggest that the TED and HMTA will more favorably bind to the Cr atoms as opposed to H_2O, with important implications for humid environments.

While the bond distance in Supplementary Table 1 decreases with stronger binding, the same trend is not seen in Supplementary Table 7. This fact can be attributed to the fundamental differences in the TED and HMTA molecules. In TED, the CH_3I sits near linearly along the N atoms of the molecule (see Supplementary Figure 7C). However, in HMTA, the nitrogen configuration is such that the open N binding sites for the CH_3I cause the CH_3I to be parallel to the organic linker of the MIL-101-Cr (see Supplementary Figure 7D). In this case, the methyl group is only 2.94 Å and 3.08 Å from the hydrogens of the organic linker and the long-range van der Waals and ionic interactions with the organic linker stabilize the molecule enough so that the longer bond distance is favorable. Iodine's high electronegativity causes a partial positive charge on the methyl group, which then interacts with and gets stabilized by the (also electronegative) benzene ring of the organic linker. In fact, the carbon of CH_3I aligns itself almost perfectly above the center and in-between the hydrogens of the linker ring. Note that this interaction is not possible in the case of CH_3I binding to TED.

(c) Transition State Study

The experimental evidence shows that upon heating from RT to 150 °C, a shift in the reaction equilibrium occurs from reactants at RT towards the products at 150 °C in

$$CH_3\text{-I} + \text{TED-MIL-101-Cr} \xrightarrow{\Delta} CH_3\text{-TED-MIL-101-Cr} + I^- .$$

On the left side, the CH_3I as a whole is bound to the TED via weak van der Waals interactions, whereas on the right side a covalent bond is formed between the methyl group and the TED while I^- is dissociated from the structure. The experimental evidence shows that both species are present at both temperatures, but at higher temperature the product configuration is dominant, suggesting that a modest barrier separates the two states. The 200 meV separation between reactants and products is low enough to expect physisorption and chemisorption at both temperatures. The 459 meV reaction barrier is consistent with the increased temperature increasing the energy of the system and allowing more CH_3I molecules to overcome the reaction barrier and chemisorb to the TED.

(d) Binding energies of possible interaction modes for I_2 with MIL-101-Cr-TED.

Binding energies were calculated for iodine species with MIL-101-Cr-TED. Possible interaction modes are depicted in Supplementary Fig. 24. All of these were optimized using the methodology described in Supplementary Note 4a and all show favorable binding (see Supplementary Table 7).

Supplementary Note 3

The kinetic studies are based on the Lagergren's pseudo-first order kinetic model by using the data during the first 0~20 min of the experiment. The nonlinear fitting is based on the equation: $q_t = q_e \times (1 - e^{-k_1 t})$, where k_1 is the kinetic constant, q_e is the adsorbed amount at equilibrium, and q_t is the amount of CH_3I adsorption at time t.

Supplementary Note 4

The high I_2 uptake was verified by sorption isotherm experiments, which gave a 44 and 42 wt% uptake for MIL-101-Cr-TED and MIL-101-Cr-HMTA at 150 °C and 150 ppm I_2 (Supplementary Fig. 21). The binding interactions between I_2 and MIL-101-Cr-TED and MIL-101-Cr-HMTA were analyzed by XPS and Raman experiments (Supplementary Figs. 22 and 23). The high I_2 uptake may be due to the formation of redox or charge transfer occurred between I_2 with N sites of TED and HMTA.[12]

Supplementary Methods

Synthesis of Ag^+@13X. Ag^+@13X was synthesized according to the previously reported procedure.[13] Typically, 1 g zeolite 13X was added into a 1 L $AgNO_3$ solution (1 M), and the mixture was stirred for 24 h and the samples were obtained by filtrate and wash with H_2O. Such exchange procedure was repeated twice to obtain the final samples. ICP: Ag: 27.4%.

Synthesis of Ag^+@ZSM-5. The same synthetic procedure for Ag^+@13X was used except that zeolite 13X was replaced by ZSM-5. ICP: Ag: 9.71%.

Synthesis of Ag^+@MOR. The same synthetic procedure as for Ag^+@13X was used except that zeolite 13X was replaced by MOR. Ag: 7.4%.

Synthesis of Ag^0@MOR. Ag^0@MOR was synthesized by hydrogenation of Ag^+@MOR under H_2 environment at 200 °C for 12 h.[14] ICP: Ag: 7.7%.

Synthesis of TED@AC (5% TED). TED (100 mg) was added into a 100 mL methanol to form a solution. Then commercial AC (1.9 g) was added into the above solution to form a slurry. The mixture was stirred for two days to evaporate all the solvent to obtain the final TED@AC.[15]

Synthesis of HMTA@AC (5% HMTA). The same synthetic procedure for TED@AC was used here except that TED was replaced by HMTA.

Synthesis of HISL. The HISL was synthesized according to the literature.[16] HISL was prepared by secondary growth using the gel consisting of TEOS, TEAOH, $(NH_4)_2SiF_6$, and H_2O using SL-1 crystals as seed crystals. The gel with a molar ratio of TEOS, TEAOH, $(NH_4)_2SiF_6$, and H_2O was 4.00 : 1.92 : 0.36 : 50 and was prepared as follows. (I) Preparation of the TEOS/TEAOH solution (Solution I): TEAOH (35%, 13.4 g) and distilled deionized water (6.3 g) were sequentially added into a plastic beaker containing 21.2 g of TEOS (98%). This beaker containing the above solution was tightly covered using the plastic wrap and magnetically stirred for about 30 min until the solution became clear. The above calcined SL-1 powder (0.24 g) was added and stirred additionally for 10 min. (II) Preparation of the TEAOH/(NH4)2SiF6 solution (SolutionII): TEAOH(35%, 6.7 g), (NH4)2SiF6 (1.64 g), and distilled deionized water (3.15 g) were introduced into a plastic beaker and stirred until all (NH4)2SiF6 became dissolved. Solution II was quickly poured into Solution I with vigorously stirring. The mixture solidified immediately. The solidified mixture was stirred for additional 2 min using a plastic rod, and aged under a static condition for 6 h. After aging, the semisolid gel was ground using a food mixer and transferred into a Teflon-lined autoclave. The sealed autoclaves were placed in an oven preheated at 165 ^oC. After 7 days, the autoclaves were removed from the oven and quickly cooled by running tap water onto them. The solid product was collected, washed with copious amounts of distilled deionized water and dried at 100 ^oC by placing them in an oven overnight. The obtained SL-1F was calcined at 550 ^oC for 16 h under oxygen flow.

Supplementary References

1. Zeng, X.-R., Ko, T.-M. Structure–conductivity relationships of iodine-doped polyaniline. *J. Polym. Sci., Part B: Polym. Phys.* **35**, 1993–2001 (1997).
2. Kalita, G., Wakita, K., Takahashi, M., Umeno, M. Iodine doping in solid precursor-based CVD growth graphene film. *J. Mater. Chem.* **21**, 15209–15213 (2011).
3. Kresse, G., Furthmüller, J. Efficient iterative schemes for ab initio total-energy calculations using a plane-wave basis set. *Phys. Rev. B* **54**, 11169–11186 (1996).
4. Kresse, G., Joubert, D. From ultrasoft pseudopotentials to the projector augmented-wave method. *Phys. Rev. B* **59**, 1758–1775 (1999).
5. Thonhauser, T. et al. Spin Signature of Nonlocal Correlation Binding in Metal-Organic Frameworks. *Phys. Rev. Lett.* **115**, 136402 (2015).
6. Berland, K. et al. Van Der Waals forces in density functional theory: a review of the vdW-DF method. *Rep. Prog. Phys.* **78**, 066501 (2015).
7. Langreth, D. C. et al. A density functional for sparse matter. *J. Phys.: Condens. Matter* **21**, 084203 (2009).
8. Thonhauser, T. et al. Van der Waals density functional: self-consistent potential and the nature of the van der Waals bond. *Phys. Rev. B* **76**, 125112 (2007).
9. Liu, K. et al. Understanding the adsorption of PFOA on MIL-101(Cr)-based anionic-exchange metal–organic frameworks: comparing DFT calculations with aqueous sorption experiments. *Environ. Sci. Technol.* **49**, 8657–8665 (2015).
10. Henkelman, G., Uberuaga, B. P., Jónsson, H. A climbing image nudged elastic band method for finding saddle points and minimum energy paths. *J. Chem. Phys.* **113**, 9901–9904 (2000).
11. Henkelman, G., Jónsson, H. Improved tangent estimate in the nudged elastic band method for finding minimum energy paths and saddle points. *J. Chem. Phys.* **113**, 9978–9985 (2000).
12. Pei, C., Ben, T., Xu S., Qiu, S. Ultrahigh iodine adsorption in porous organic frameworks, *J. Mater. Chem. A*, **2014**, *2*, 7179–7187.
13. Aguado, S. et al. Absolute molecular sieve separation of ethylene/ethane mixtures with silver Zeolite A. *J. Am. Chem. Soc.* **134**, 14635–14637 (2012).
14. Chapman, K. W., Chupas, P. J., Nenoff, T. M. Radioactive iodine capture in silver-containing Mordenites through nanoscale silver iodide formation. *J. Am. Chem. Soc.* **132**, 8897–8899 (2010).
15. Shah, I., Adnan, R., Wan Ngah, W. S., Mohamed, N. Iron impregnated activated carbon as an efficient adsorbent for the removal of methylene blue: regeneration and kinetics studies. *PLoS ONE* **10**, e0122603 (2015).
16. Pham, T. C. T. *et al.* Capture of iodine and organic iodides using silica zeolites and the semiconductor behaviour of iodine in a silica zeolite. *Energy Environ. Sci.* **9**, 1050–1062 (2016).
